# Modulating the interfacial properties of rice protein to enhance its applicability as an emulsifier and foaming agent in the food industry

**DOI:** 10.1016/j.fochx.2026.104554

**Published:** 2026-10-03

**Authors:** Zhuangpeng Wang, Xinwen Huang, Yu Zhao, Leiyan Wu

**Affiliations:** a College of Agriculture, Jiangxi Agricultural University, Nanchang 330045, China; b Ministry of Education and Jiangxi Key Laboratory of Crop Physiology, Ecology and Genetic Breeding, Jiangxi Agricultural University, Nanchang 330045, China; c College of Food Science and Engineering, Jiangxi Agricultural University, Nanchang 330045, China

**Keywords:** Rice protein, Interface adsorption, Applicability, Emulsifier, Foaming agent

## Abstract

Interfacial adsorption of proteins underlies their emulsifying and foaming properties. However, the rigid molecular structure of natural rice protein severely limits its application as a functional emulsifier and foaming agent. Although various modification strategies have been proposed, comprehensive reviews that systematically elucidate the mechanisms improving rice protein's emulsifying and foaming properties from the interfacial adsorption perspective are scarce. This review systematically summarizes key strategies and the practical challenges to enhance rice protein's interfacial properties, clarifies the relationship between interfacial adsorption and emulsifying/foaming performance, evaluates its potential as a plant-based emulsifier and foaming agent in the food industry, and proposes future research directions for precise regulation of its interfacial adsorption behavior. This review is expected to fill the knowledge gap on the dynamic relationships among rice protein's molecular structure, interfacial adsorption, and emulsifying and foaming properties, thereby providing a theoretical basis for improving the quality of complex multiphase food systems.

## Highlights

• Synthesized the root cause of poor interface activity in rice protein from the inside structure.
• Summarized the relationship between interfacial adsorption, emulsifying and foaming properties.
• Analyzed the application potential of modified rice protein in the food industry.
• Elaborated on the frontier directions to enhance the interface adsorption of rice protein.

## 1 Introduction

By 2050, the global population is projected to exceed 9 billion (Kim et al., 2024; Smith et al., 2024). Against this backdrop, the sustainable supply of high-quality protein has become one of the central challenges facing the global food system. While animal-based proteins offer significant advantages in amino acid composition, digestibility, absorption rate, and functional properties, their large-scale production entails substantial water consumption, greenhouse gas emissions, and ethical concerns regarding animal welfare—limiting their long-term environmental sustainability (Sampedro, 2024). In contrast, plant-based proteins such as rice protein, soy protein, pea protein, and wheat protein possess inherent advantages such as diverse raw material sources, short growing cycles, and high land and water use efficiency per unit of protein produced, increasingly positioning them as a key strategic resource for building resilient, low-carbon, and inclusive food systems (Inanlar & Capanoglu, 2026). Among these, rice protein has garnered growing attention due to its distinct benefits. Compared to soy protein, rice protein exhibits notably low allergenicity, making it an ideal choice for infant formula, individuals with allergies, and vegetarians. Additionally, unlike wheat protein, rice protein is naturally gluten-free, providing a critical alternative for consumers with gluten intolerance (Chen et al., 2023; Jayaprakash et al., 2022; Roy et al., 2025).

However, natural rice protein typically exhibits low interfacial activity, weak interfacial adsorption capacity, and a sparse, disordered interfacial adsorption layer. These inherent limitations can be attributed to three primary factors: I. Hydrophobicity and aggregation. Rice protein, particularly its major component glutelin, is classified as a hydrophobic protein. However, in its native conformation, hydrophobic regions are often buried within the protein structure, limiting their exposure at the oil–water interface (Zhu et al., 2024). Furthermore, rice protein has relatively low solubility, which significantly impairs its dispersibility in aqueous systems and its ability to diffuse toward interfaces (Wang et al., 2018; Yang et al., 2023a). For example, studies have reported that the solubility of native rice protein may be as low as 1.84% (Wang et al., 2018). This limited solubility promotes protein aggregation in solution, leading to the formation of larger particles, which in turn reduces the rate and efficiency of interfacial adsorption (Shen, Dai, et al., 2023; Yang et al., 2023b). II. Molecular weight and structural flexibility. Rice protein possesses a relatively high molecular weight and a rigid molecular structure, which hinders its ability to rapidly unfold and reorient upon adsorption at the interface (Li et al., 2023; Zhao et al., 2020). The unfolding and reorganization of protein molecules at the interface are critical for the formation of a stable interfacial film. Any impairment in this process directly compromises its functionality as an emulsifier and foaming agent (Wang, Chen, et al., 2025; Wierenga et al., 2009). III. Charge characteristics. The isoelectric point (pI) of a protein determines its net charge under varying pH conditions. For rice protein, the pI typically ranges between pH 4 and 5. At this pH range, the net charge approaches zero, resulting in reduced electrostatic repulsion between protein molecules. This facilitates aggregation and precipitation, further diminishing solubility and interfacial adsorption capacity (Mun et al., 2020; Xu et al., 2017). Therefore, the development of effective strategies to enhance the interfacial properties of rice protein is essential to improve its functional performance and expand its application as a plant-based emulsifier and foaming agent in the food industry.

Currently, extensive research has been conducted on the functional modification of rice protein across diverse fields (as depicted in Fig. 1, Fig. 2), aimed at enhancing its solubility, interfacial activity, and adsorption capacity at oil-water or air-water interfaces. The data in Fig. 1, Fig. 2 are sourced from the web of science core collection, centered on topics pertaining to modified rice protein or interfacial properties. The publication years span from 2020 to 2026, with the document type restricted to articles. The subject categories include all fields (6465 entries) and food science technology (870 entries). These efforts aim to meet the growing demand of the modern food industry for sustainable plant-based protein ingredients. However, comprehensive reviews that systematically elaborate on the mechanisms underlying the improvement of rice protein's emulsifying and foaming properties from the perspective of interfacial adsorption remain scarce. In particular, the dynamic relationships between rice protein's molecular structure, interfacial adsorption, and emulsion/foam stability have not yet been systematically revealed and theoretically integrated. To address this gap, this review focuses on analyzing the regulatory rules of rice protein's molecular structural characteristics on its interfacial adsorption behavior, and based on the essence of the interfacial adsorption process, systematically clarifies the structure-function relationships between interfacial adsorption and the emulsifying and foaming functionalities of the protein. Overall, this review strives to provide a solid theoretical foundation for the application of rice protein as a novel plant-based emulsifier and foaming agent in food products, and to promote its industrial development and utilization. (See Table 1, Table 2.)

**Fig. 1:**
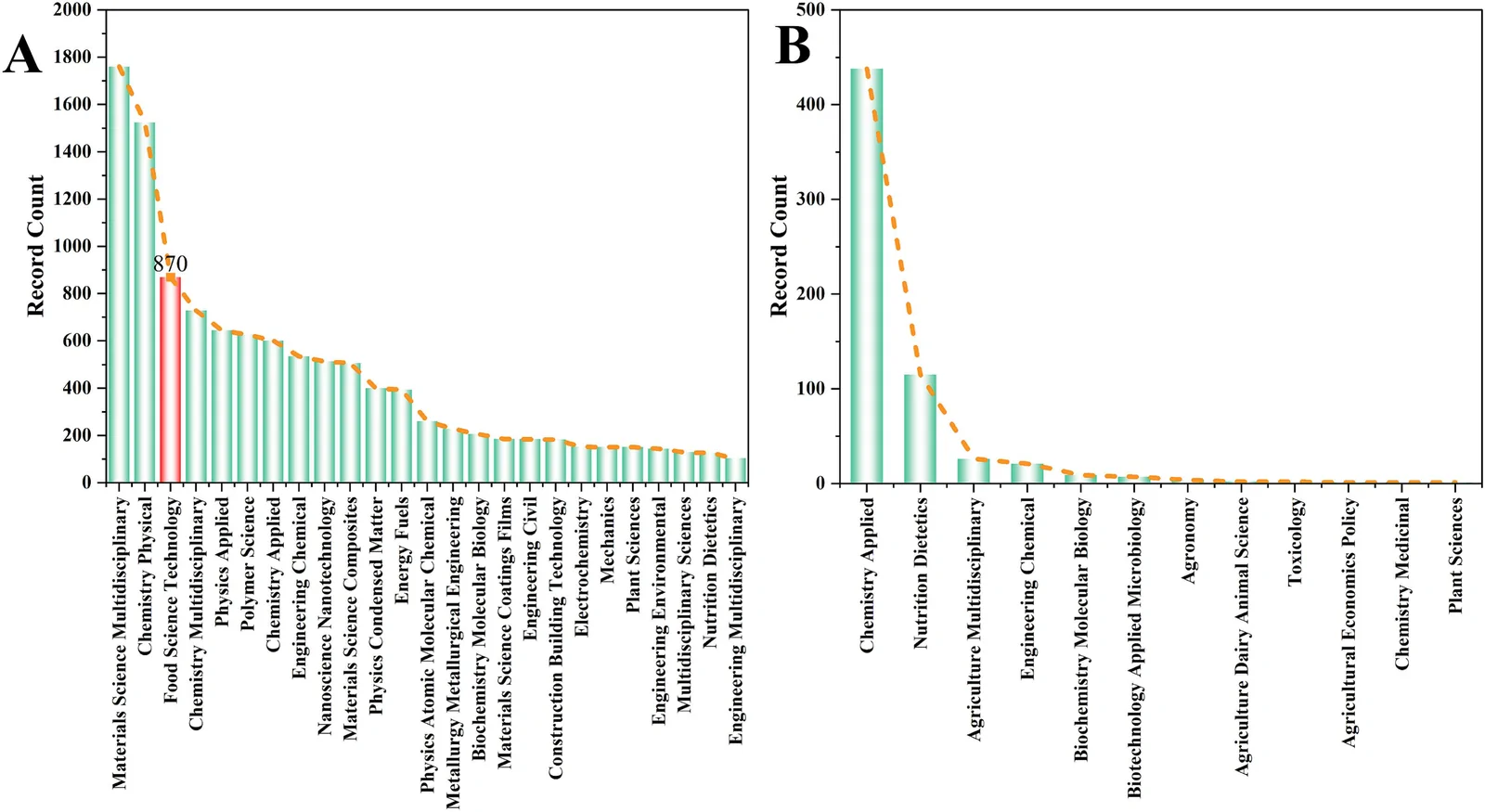
The number of literatures on modified rice protein and interfacial properties searched on the Web of Science website from 2020 to 2026. Among them, Fig. 1A includes all fields, and Fig. 1B includes the field of food science technology.

**Fig. 2:**
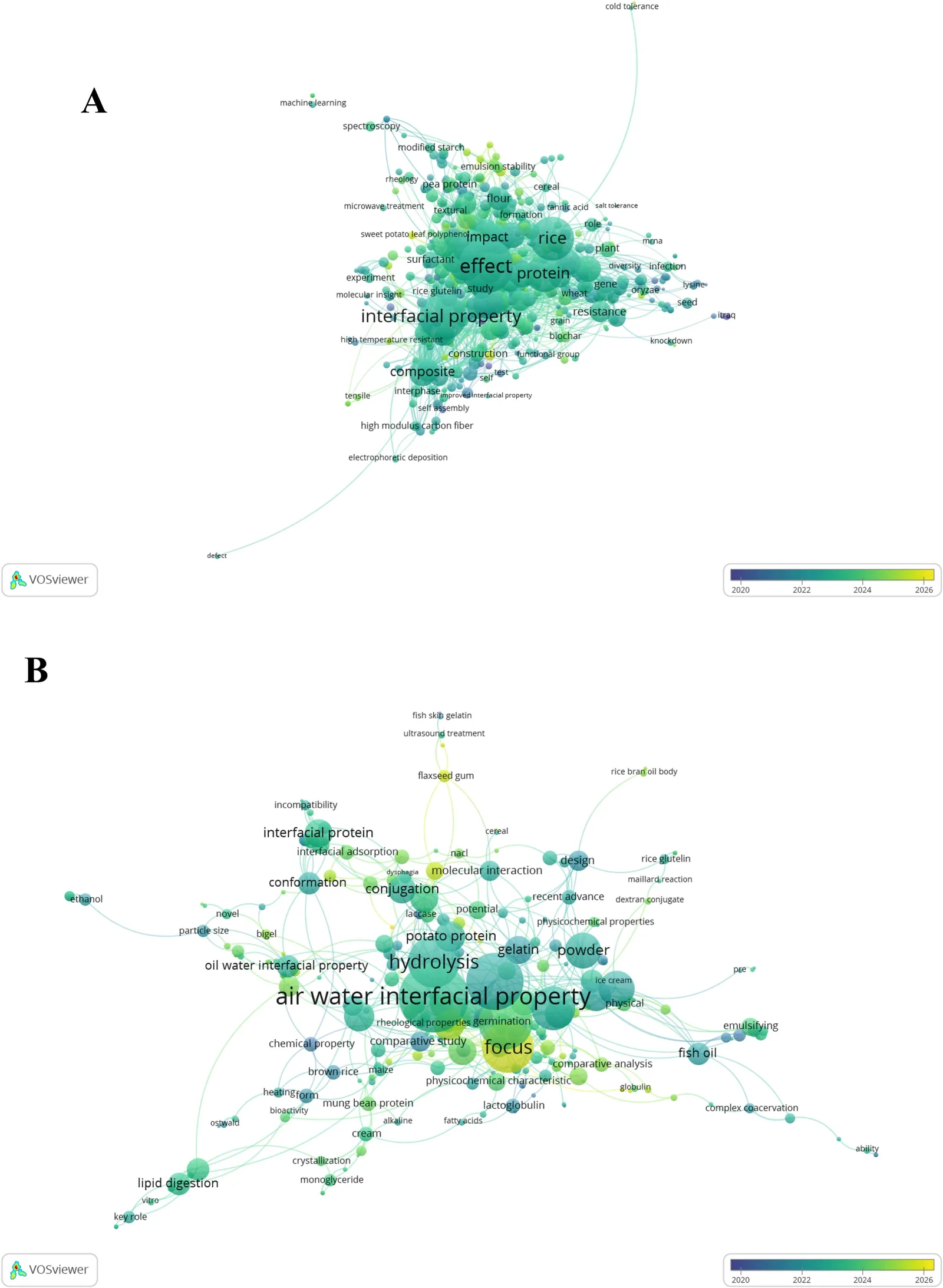
Visualization map of research hotspots on the interfacial properties of modified rice protein from 2020 to 2026. Among them, Fig. 2A covers research hotspots across all fields, and Fig. 2B focuses on those within the field of food science technology.

**Table 1:** Overview of key studies on the enhancement of the interfacial properties of rice protein through physical, chemical, enzymatic, and combined modification strategies from 2019 to 2025.

Modification strategies	Rice cultivars	Types of rice protein	pH	protein concentration	oil-phase composition	Homogenization speed	homogenization time	Enhancement effects	Possible reasons	References
Ultrasound	Ning Xiang Geng No.9	Rice protein	pH 7.0	1%, *w*/*v*	Soybean oil	20,000 rpm	2 min	Foaming capacity (189.5%) Emulsifying capacity (77.1 mL/g)	Particle size ↓ Zeta-potential ↑ Solubility ↑	(Zhang, Zheng, et al., 2025)
Ultrasound	–	Rice bran protein	pH 7.0	10 mg/mL	Soybean oil	15,000 rpm	2 min	Emulsion stability (336.43 min)	Zeta-potential ↑ Surface hydrophobicity ↑ Conformational flexibility ↑	(Liu et al., 2024)
High hydrostatic pressure	–	Rice bran protein	pH 7.0	5 mg/mL	Soybean oil	10,000 rpm	1 min	Emulsifying activity (31.82 m^2^/g) Emulsion stability (24.69 min)	Particle size ↓ Zeta-potential ↓	(Wang, Li, et al., 2025)
Extrusion	–	Rice protein	pH 7.0	10 mg/mL	Corn oil	20,000 rpm	1 min	Emulsion stability (152.82%)	Sulfhydryl groups ↓ Disulfide bonds ↓ Hydrogen bond ↑ Ionic bonds ↓ Hydrophobic effect ↓	(Gao et al., 2022)
pH shift	–	Rice protein isolates	pH 7.0	1%, w/v	Sunflower oil	10,000 rpm	1 min	Emulsifying activity (49.5 m^2^/g) Emulsion stability (59.5 min)	Permeation rate ↑ Rearrangement rate ↑ Particle size ↓	(Shen, Zheng, et al., 2023)
Heating	–	Rice glutelin	pH 12.0	2 mg/mL	–	8000 rpm	1 min	Foaming capacity (≈126.00%)	Particle size ↓ Disulfide bond ↓ Surface net charge ↑ Surface hydrophobicity ↑	(Zhao et al., 2020)
Heating	–	Rice bran protein	pH 7.4	1 mg/mL	Soybean oil	10,000 rpm	1 min	Emulsion stability (≈700 min)	*β*-sheet structure ↑ Interfacial activity ↑ Surface hydrophobicity ↑	(Luo et al., 2025)
Glycosylation	–	Rice protein	pH 8.0	2%, w/v	–	11,000 g	40 s	Foaming ability (180.31%) Foam stability (65.10%)	Surface hydrophobicity ↑ Solubility ↑	(Cheng et al., 2021)
Glycosylation	Fengyou indica rice	Rice protein	pH 7.0	2%, w/v	Soybean oil	10,000 rpm	30 s	Emulsifying activity (0.899) Emulsion stability (216.68 min)	Surface hydrophobicity ↓ Solubility ↑	(Cheng et al., 2022)
Deamidation	–	Rice protein	pH 7.0	1 mg/mL	Corn oil	12,000 rpm	2 min	Emulsifying capacity (44.19 m^2^/g)	Solubility ↑	(Shi, Xie, Ouyang, Shi, et al., 2024)
Phosphorylation	Glutinous rice	Rice glutelin	pH 7.0	1 mg/mL	Sunflower seed oil	10,000 rpm	1 min	Emulsifying capacity (29.4 m^2^/g)	Particle size ↓ Zeta-potential ↑	(Wang et al., 2019)
Acylation	–	Rice glutelin	pH 7.0	1%, w/v	–	10,000 rpm	3 min	Foaming capacity (102.44%) Foam stability (97.59%)	Particle size ↓ Zeta-potential ↑ Solubility ↑	(Luo et al., 2024)
Enzymatic hydrolysis	–	Rice protein	pH 7.0	1%, w/v	Corn oil	12,000 rpm	3 min	Emulsifying capacity (71.45 m^2^/g)	Solubility ↑ Conformational flexibility ↑	(Qi, Li, et al., 2024)
Transglutaminase cross-linking	Indica rice	Rice bran protein	pH 7.0	30 mg/mL	–	–	1.5 min	Foaming capacity (375%) Foam stability (79.45%)	Particle size ↓ Zeta-potential ↓ Interfacial elastic modulus ↑	(Wu et al., 2023)
Ultrasonic combined with alkali heat treatment	–	Rice protein	pH 7.0	10 mg/mL	Rice bran oil	10,000 rpm	2 min	Emulsion stability (≈360.00 min)	Particle size ↓ Wettability ↑ Random coil structure↑	(Zhu et al., 2025)
pH shift combined with ion induction	–	Rice dreg protein	pH 7.0	20 mg/mL	–	10,000 rpm	1 min	Foaming capacity (300.00%) Foam stability (73.00%)	Particle size ↓ Conformational flexibility ↑ Interface adsorption ↑ Interfacial film ↑	(Yang, Chen, et al., 2024)
Pea protein and rice bran protein interaction	–	Rice bran protein	pH 7.0	1.25 mg/ml	Palm oil	10,000 rpm	1 min	Emulsion stability (≈145%)	Continuous phase viscosity ↑ Interfacial tension ↓	(Tong et al., 2024)

–: Indicates unspecified

**Table 2:** The comparison of mechanisms, pros and cons, applicable scenarios, and intrinsic limitations of physical, chemical, enzymatic, and composite modification.

Modification strategies	Modification mechanisms	Pros	Cons	Applicable scenarios	Intrinsic limitations
Ultrasound	Disruption of non-covalent bonds; partial unfolding of proteins	Short processing time; low temperature; minimal nutrient loss	Limited penetration depth; high equipment cost	Fresh-keeping; protein modification	Energy conversion efficiency low; scale-up difficult
High hydrostatic pressure	Protein unfolding and reconfiguration	Low temperature operation; uniform processing; preserves nutrients	Expensive equipment; limited product size	Meat tenderizer; dairy products	Pressure-resistant equipment required
Extrusion	High temperature and shear forces cause protein melting and restructuring	Continuous production; versatile product shapes; cost-effective	High energy consumption; potential nutrient loss	Snack foods; textured vegetable protein	Equipment wear; limited to low moisture products
pH shift	Adjusting pH away from protein isoelectric point causes unfolding	Simple operation; low temperature processing; improves solubility	Difficult pH control; bitter taste	Tofu production; protein gel preparation	Requires a precise pH control system
Heating	Heat causes protein denaturation and structural reorganization	Well-established process; improves gel properties	Nutrient degradation; may cause over-denaturation	Cooked meats; bakery products; gels	Limited control over reaction pathways
Glycosylation	Reducing sugars react with amino groups via Maillard reaction	Improves emulsification; enhances flavor; water binding	May reduce nutritional value; complex reaction control	Baked goods; processed meats; flavoring agents	Require reducing sugar; may produce melanoidins
Deamidation	Removal of amide group converts glutamine/asparagine to acids	Improves solubility and emulsification; enhances protein functionality	May alter protein structure unexpectedly; limited specificity	Protein supplements; functional beverages	Require acid/alkali treatment; potential nutrient loss
Phosphorylation	Adding phosphate groups via kinase or chemical phosphorylation	Improves thermal stability; enhances emulsification	Chemical reagents required; potential safety concerns	Dairy products; sports supplements	Reagent cost; regulatory approval needed
Acylation	Adding acyl groups (acetyl, succinyl) to amino acid side chains	Improves solubility at various pH; enhances emulsification	May reduce protein digestibility; reagent cost	Protein supplements; vegetarian products	Require acylating agents; potential allergenicity
Enzymatic hydrolysis	Proteases break peptide bonds into smaller peptides	Produces bioactive peptides; improves digestibility	Difficult to control degree of hydrolysis; bitter taste	Infant formula; sports nutrition; functional foods	Enzyme cost; bitter peptide formation
Transglutaminase cross-linking	TGase catalyzes isopeptide bonds between glutamine and lysine	Improves gel strength; creates elastic texture	Expensive enzyme; may create unfamiliar peptides	Surimi; cheese; bakery products	Require Ca^2+^; narrow pH optimum
Ultrasonic combined with alkali heat treatment	Ultrasound disrupts structure; alkali heat promotes unfolding	Synergistic effect; efficient protein unfolding	Complex process; difficult parameter optimization	Plant protein modification; gel preparation	Process control complexity; high energy input
pH shift combined with ion induction	pH shift exposes groups; ions (Ca^2+^, Zn^2+^) induce aggregation	Enhanced gelation; improved texture	Ion interference; limited ion types	Calcium-fortified foods; protein gels	Ion specificity; mineral interactions
Pea protein and rice bran protein interaction	Non-covalent interactions and enzymatic cross-linking	Complementary amino acid profiles; improved nutrition	Interaction complexity; variability in raw materials	Plant-based meat alternatives; nutritional supplements	Raw material variability; extraction cost

## 2 Structural characteristics of rice protein

As a significant plant-based protein source, rice protein exhibits structural characteristics that exert a decisive influence on its interfacial properties. Elucidating its molecular structure is fundamental to optimizing interfacial adsorption behavior and broadening its functional applications in the food industry.

### 2.1 Composition and classification of rice protein

According to the Osborne classification system, which categorizes proteins based on their solubility in different solvents, rice proteins can be classified into four major groups: albumin (water-soluble), globulin (salt-soluble), prolamin (alcohol-soluble), and glutelin (acid or alkali-soluble) (Ghanghas et al., 2020; Zhang, Wang, Li, et al., 2024). Among these, rice glutelin serves as the primary storage protein, accounting for approximately 80% of the total protein content. It is characterized by a balanced amino acid profile, high nutritional value and low allergenic potential, thereby offering significant potential for application in the food industry (Wang, Ettelaie, & Sarkar, 2025). However, despite its nutritional benefits, the widespread use of rice glutelin in food processing is hindered by its limited solubility (Luo et al., 2024; Yang et al., 2023a). This restriction is primarily attributed to the presence of large molecular weight fragments interconnected by disulfide bonds, which tend to aggregate and result in poor water solubility (Jiang et al., 2024; Zhu et al., 2024). Rice globulin represents another key storage protein in rice seeds and, together with glutelin, prolamin, and albumin, contributes to the intricate protein composition of rice (Jayaprakash et al., 2022). Research has demonstrated that rice globulin has an approximate molecular weight of 26,000 Da and an isoelectric point of 6.4. It exhibits a degree of sequence homology with wheat gliadin and is rich in glycine and glutamic acid/glutamine, although it contains relatively low levels of lysine (Hibino et al., 1989; Komatsu & Hirano, 1992). Rice albumin is a notable water-soluble protein found in rice. Compared to other major storage proteins such as glutelin, prolamin, and globulin, rice albumin constitutes only a small proportion of total rice protein, approximately 5% (Mawal et al., 1987). Nevertheless, its biological functions and potential applications have garnered increasing scientific interest in recent years (Jayaprakash et al., 2022; Kadowaki et al., 2019). Rice prolamin is an essential storage protein belonging to the cereal prolamin family. It is defined by its solubility in 60% (*v*/v) isopropanol and other alcoholic solvents (Ruan et al., 2020). This protein group primarily consists of polypeptide subunits with molecular weights of approximately 10, 13, and 16 kDa. Its amino acid composition is marked by high levels of glutamic acid/glutamine and leucine, but it is relatively deficient in lysine—a characteristic commonly observed among prolamins from various cereal grains (Hibino et al., 1989; Pham et al., 2024).

It is worth noting that the actual composition and classification of rice proteins are not fixed, as they are influenced by multiple factors, with rice cultivars and raw material sources being the most prominent ones. Genotypic differences among various rice cultivars (such as indica, japonica, and glutinous rice) lead to significant variations in the relative proportions of protein fractions (albumin, globulin, prolamin, and glutelin) (Xinkang et al., 2023). For example, indica and japonica cultivars with high amylose content or specific eating quality exhibit systematic differences in the ratio of glutelin to prolamin; these differences not only affect the eating quality of cooked rice but also determine the functional properties of extracted proteins (Liang et al., 2022; Ma et al., 2024). Agronomic management practices like nitrogen fertilizer application may further regulate the relative contents of these fractions. Studies have shown that under different nitrogen levels, the contents of glutelin and globulin change significantly, which in turn affects the secondary structure and functional performance of proteins (Liang et al., 2022). In addition, raw material sources (e.g., whole rice, rice bran, and other components) are another key variable determining the composition and classification of rice proteins. As a by-product of rice milling, rice bran is rich in albumin and globulin, and its protein composition is distinctly different from the glutelin-dominant proteins in the endosperm (Fabian & Ju, 2011). Rice bran protein typically contains high proportions of water-soluble and salt-soluble fractions, but due to the accompanying large amounts of lipids, dietary fiber, and phenolic compounds, the purity and aggregation state of its extracts are extremely complex (Rivero Meza et al., 2024). In contrast, proteins extracted from whole rice mainly reflect the characteristics of endosperm storage proteins, which often undergo irreversible aggregation and disulfide bond cross-linking during industrial extraction processes such as alkaline extraction and acid precipitation, forming high-molecular-weight, rigid aggregates (Yang et al., 2023b). These differences in accompanying components and aggregation states, determined by cultivars and raw material sources, directly affect the structural characteristics and interfacial properties of protein molecules. Therefore, when evaluating and applying rice proteins as functional food ingredients, it is essential to fully consider the inherent differences in subunit composition and structure caused by their cultivars and raw material sources. This is crucial for precisely regulating their interfacial adsorption behavior and optimizing their applications in the food industry.

### 2.2 Molecular structure of rice protein

In the food industry, the manifestation of functional properties such as emulsification and foaming is predominantly governed by the molecular structure of proteins and their conformational dynamics. Therefore, a comprehensive understanding of the primary structure and higher-order structural features of rice protein is essential for the effective utilization and optimization of its functional attributes. The molecular architecture of a protein originates from its amino acid sequence, referred to as the primary structure (Clark et al., 2019), which fundamentally dictates the formation of subsequent higher-order structures and associated functional behaviors (Fig. 3) (Baland et al., 2024; Helmick et al., 2023; Macedo & Melo, 2024). Protein secondary structure refers to the recurring local folding patterns formed along the polypeptide backbone, primarily consisting of *α-*helices, *β*-sheets, and random coils. The *α*-helix is a right-handed helical conformation stabilized by intrachain hydrogen bonds, whereas the *β*-sheet is composed of multiple peptide strands arranged either in parallel or antiparallel orientations and interconnected via interstrand hydrogen bonds (Alrosan et al., 2024; Yamamoto et al., 2024). The tertiary structure describes the three-dimensional folded conformation of a single polypeptide chain, while the quaternary structure pertains to the supramolecular assembly of two or more polypeptide subunits, stabilized by non-covalent or covalent interactions. These higher-order structural arrangements are critical determinants of protein functionality, primarily governed by a range of side-chain interactions, including hydrophobic effects, hydrogen bonding, ionic interactions, and disulfide linkages (Alrosan et al., 2024; Baland et al., 2024; Clark et al., 2019; Janin et al., 2008; Khan et al., 2023; Peyravi et al., 2018; Socci, 1992). For rice protein, especially its main component glutelin, it possesses a high molecular weight, a rigid three-dimensional conformation, and an extensive disulfide bond cross-linking network (Zhao et al., 2020). While these structural features endow it with excellent conformational stability in aqueous systems, they significantly restrict its rapid adsorption kinetics and interface-induced conformational rearrangement ability at oil-water or air-water interfaces (Amagliani et al., 2017a). The adsorption of proteins at interfaces is inherently an energy-driven process: to reduce interfacial free energy, molecules must undergo directional unfolding, exposing hydrophobic groups to the non-polar phase (oil or gas phase) and orienting hydrophilic groups toward the aqueous phase (Kontogiorgos & Prakash, 2023; Tang & Shen, 2015). However, the dense disulfide bond cross-linking and low-flexibility backbone in glutelin collectively increase the activation energy barrier required for conformational rearrangement. This makes it difficult for glutelin to complete sufficient unfolding and ordered rearrangement within the interfacial residence time, thereby hindering the formation of a continuous, dense, and mechanically robust interfacial film (Yang et al., 2022). This restricted interfacial conformational dynamics is the fundamental structural root cause for the usually low emulsification stability and foaming capacity of natural rice protein.

**Fig. 3:**
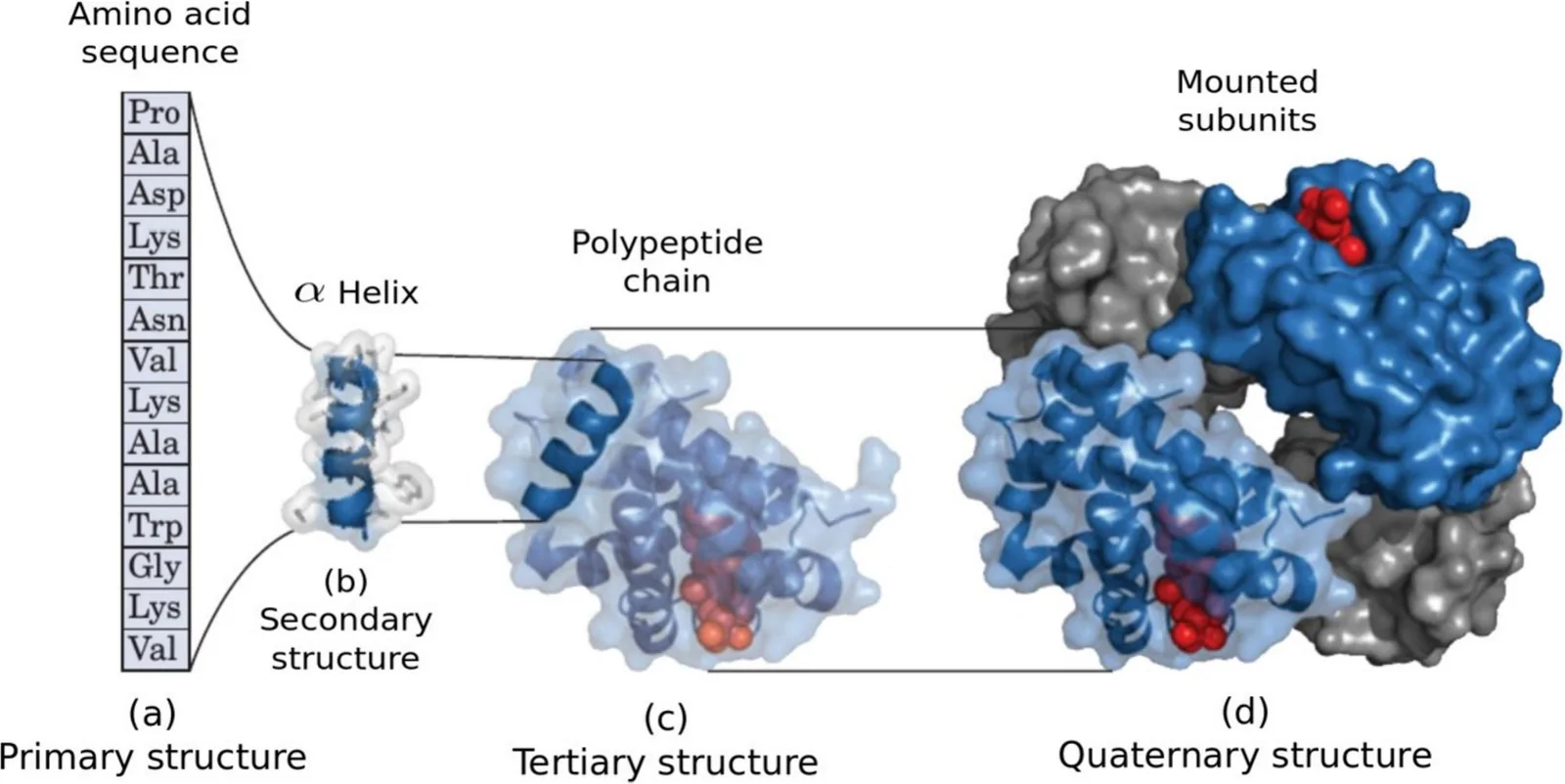
Hierarchy of protein structures at four levels (Macedo & Melo, 2024).

### 2.3 The amino acid composition and nutritional value of rice protein

Rice protein, with its unique amino acid composition and excellent nutritional value, has become an important component of plant protein sources and has shown significant potential in meeting the growing demand for plant-based proteins. The amino acid composition is the core factor determining its nutritional value. Numerous studies have shown that rice protein contains all essential amino acids for the human body and has high nutritional value, making it a high-quality plant protein source (Kalman, 2014; Kawakatsu & Takaiwa, 2019). However, the distribution of its essential amino acids is not entirely balanced. It has relatively high levels of glutamic acid/glutamine and leucine, but a relatively low level of lysine (Hibino et al., 1989; Kalman, 2014). For instance, the analysis of organic whole-grain brown rice protein concentrates and isolate revealed that the lysine content was 2.4 g and 2.2 g per 100 g of protein, respectively, significantly lower than that of whey protein isolate (9.7 g) and soy protein isolate (6.0 g) (Kalman, 2014). This characteristic is similar to that of numerous cereal proteins, indicating that lysine is typically the limiting amino acid (Khoi et al., 1987). Moreover, different rice varieties, grain parts, and processing methods can all affect its amino acid composition, thereby further influencing its nutritional quality.

## 3 Key factors governing the interfacial properties of rice protein

As amphiphilic surface-active molecules, proteins can adsorb at oil–water or air–water interfaces, reduce interfacial tension, and stabilize emulsion or foam systems by forming dense, viscoelastic interfacial films. However, the interfacial activity of natural rice proteins is relatively low, and their emulsifying and foaming properties are significantly constrained by multiple intrinsic and environmental factors. Intrinsic factors mainly include the conformational flexibility, surface hydrophobicity, solubility, and charge distribution of proteins; environmental factors cover key parameters such as pH, ionic strength, protein concentration, and temperature (Wu et al., 2021; Yang et al., 2023a; Zhang, Diao, et al., 2022).

### 3.1 Conformational flexibility

The molecular structure of proteins, particularly their rigidity and conformational flexibility, exerts a decisive influence on their adsorption behavior at oil–water or air–water interfaces and subsequent interfacial film formation (Zhang, Diao, et al., 2022). Natural rice proteins typically possess a high molecular weight and rigid conformation, resulting in slow unfolding and rearrangement kinetics during interfacial adsorption, which hinders the timely exposure of hydrophobic groups. This structural rigidity significantly delays the initial adsorption rate, as the protein must overcome a large conformational energy barrier to achieve full unfolding and exposure of hydrophobic domains (Kondo et al., 1992; Saikia et al., 2014). In contrast, proteins with higher conformational flexibility can unfold rapidly at the interface, exposing internal hydrophobic regions more effectively, thereby facilitating efficient adsorption, tight packing, and the formation of stable interfacial films (Jin et al., 2023; Liang & Tang, 2013; Tang and Shen, 2013, Tang and Shen, 2015), this rapid interfacial response is essential for the initial formation and stabilization of both emulsions and foams.

### 3.2 Surface hydrophobicity

Hydrophobicity is one of the core physicochemical parameters governing the interfacial adsorption behavior of rice proteins at oil–water or air–water interfaces. This adsorption process encompasses multiple kinetic stages, including diffusion, conformational unfolding, and molecular rearrangement (Fig. 4), which collectively determine the macroscopic stability of the resulting emulsion and foam systems (Miller et al., 2000; Wierenga et al., 2003; Yu et al., 2023; Zhai et al., 2013). As amphiphilic macromolecules, rice proteins contain both hydrophilic and hydrophobic amino acid residues in their molecular structure. Upon adsorption at the interface, the hydrophobic segments orient toward the non-aqueous phase (air or oil), while the hydrophilic regions remain in the aqueous phase, thereby reducing interfacial tension and forming a protective barrier (Miller et al., 2000; Zhang, Fan, et al., 2022). However, the hydrophobic regions of natural rice proteins are usually buried within the protein structure, limiting their exposure at the oil–water interface. Additionally, excessively high overall hydrophobicity may induce uncontrolled aggregation of proteins in the aqueous phase, impairing their solubility and interfacial accessibility, and thus hindering adsorption kinetics and interfacial film formation (Delahaije & Wierenga, 2022). Therefore, moderate surface hydrophobicity—defined as the effective exposure of hydrophobic groups on the molecular surface—is a prerequisite for achieving efficient interfacial adsorption and stabilizing emulsion and foam systems.

**Fig. 4:**
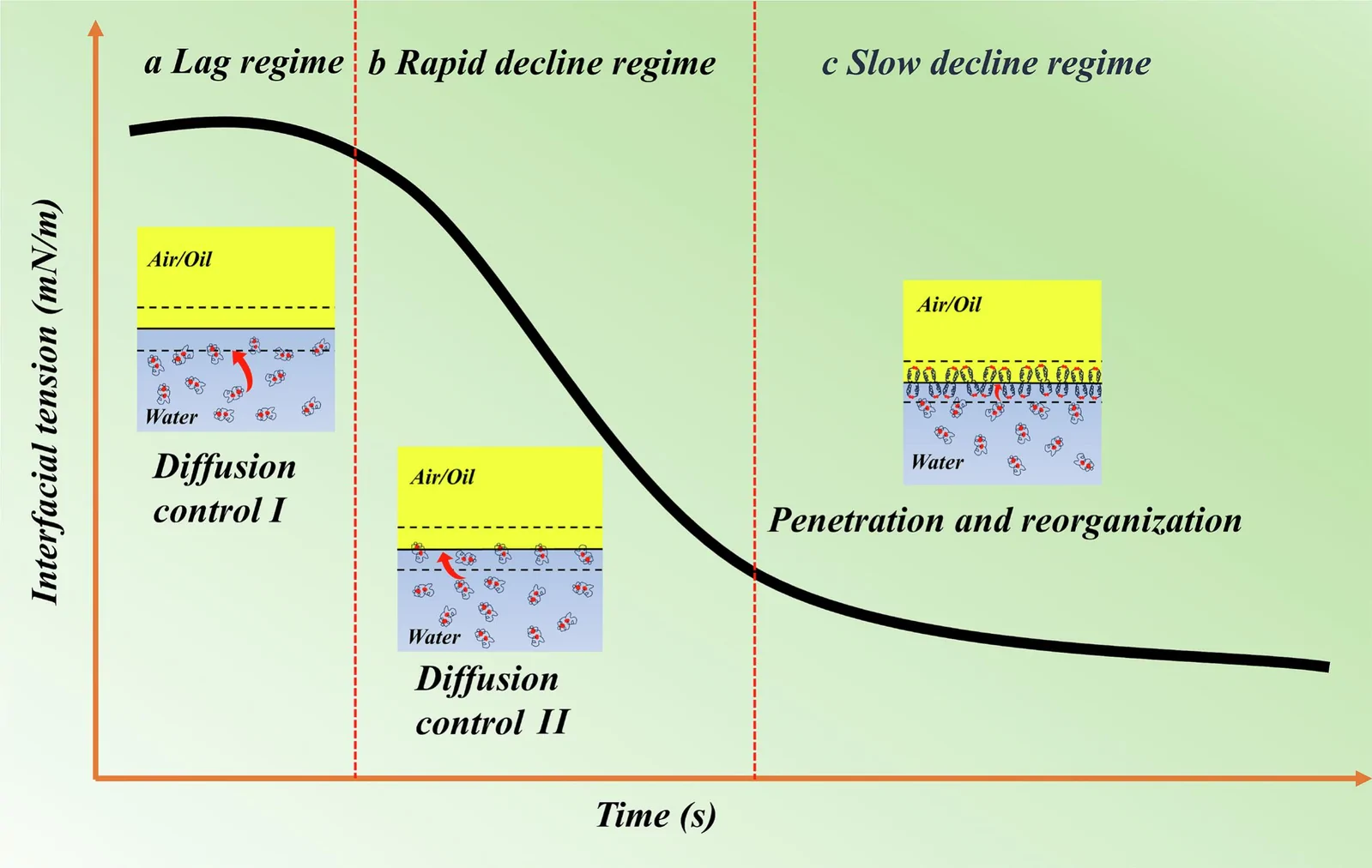
Typical dynamic interfacial tension changes in protein adsorption at the air/oil-water interface over time.

### 3.3 Solubility

Solubility is a key physicochemical parameter determining the interfacial behavior of rice proteins. Natural rice proteins usually exhibit low water solubility, which severely restricts their dispersion uniformity in aqueous systems and their ability to diffuse and migrate to oil–water or air–water interfaces (Zhao et al., 2020). Low solubility tends to induce intermolecular aggregation of proteins, forming large-sized aggregates or rigid supramolecular structures, thereby significantly reducing their interfacial diffusion rate, adsorption efficiency, and conformational rearrangement capacity (Jiang et al., 2024; Manzoor et al., 2022; Wang, Hua, et al., 2025). The resulting interfacial films commonly suffer from poor continuity, uneven thickness, insufficient mechanical strength, and inadequate elasticity, making them unable to effectively stabilize long-lasting emulsion or foam systems (Mitropoulos et al., 2014; Zhong et al., 2024). In contrast, well soluble proteins typically possess superior hydration capacity and colloidal stability, and tend to exist as monomers or metastable small aggregates (Jiang et al., 2024; Zheng et al., 2024). This ensures rapid interfacial diffusion, sufficient molecular unfolding, and ordered arrangement, ultimately forming dense, continuous interfacial films with both high mechanical strength and excellent elasticity.

### 3.4 Charge characteristics

The net charge of proteins is a key electrochemical parameter regulating their interfacial adsorption behavior (Perriman & White, 2006; Sengupta et al., 1999). When the system pH deviates significantly from the pI of rice proteins, the protein surface carries a considerable net charge, enhancing intermolecular electrostatic repulsion and thus favoring the maintenance of excellent colloidal dispersibility and aqueous phase stability (Cho et al., 2022; Xu et al., 2017). However, under conditions close to the pI, the net charge of the protein approaches zero, leading to a substantial reduction in electrostatic repulsion and easily inducing intermolecular aggregation and even irreversible precipitation (Xu et al., 2016; Xu et al., 2017). This phenomenon not only significantly reduces water solubility and interfacial accessibility but also disrupts adsorption kinetics: while a low net charge state may shorten the diffusion path and increase interfacial migration rate, the resulting interfacial film often lacks sufficient compactness, mechanical strength, and elastic responsiveness due to the absence of adequate intermolecular electrostatic stabilization, making it difficult to effectively support the long-term stability of emulsions or foams (Shen, Dai, et al., 2023; Xu et al., 2016).

### 3.5 Environmental factors

Environmental factors exert significant influence on the effective adsorption of rice protein at two-phase interfaces, particularly with respect to pH, ionic strength, protein concentration, and temperature. These parameters modulate the structural conformation, charge distribution, and intermolecular interactions of rice protein, thereby affecting its interfacial adsorption kinetics, the formation of interfacial films, and the overall stability of emulsions and foams (Abd Rahim et al., 2024; Hao et al., 2024; Luo et al., 2025; Luo, Deng, et al., 2023; Romero et al., 2012; Shen, Dai, et al., 2023; Wu et al., 2023).

#### 3.5.1 pH

The regulation of pH conditions can significantly alter the net charge state, conformational flexibility, and intermolecular interaction strength of rice protein, thereby profoundly influencing its dynamic adsorption behavior at air-water and oil-water interfaces. As a plant-derived emulsifier and foaming agent with important application prospects in the food industry, the interfacial adsorption process of rice protein typically involves three consecutive stages: (I) diffusion of the protein from the bulk phase to the interface; (II) conformational rearrangement at the interface post-adsorption to expose internal hydrophobic groups; and (III) formation of a viscoelastic interfacial film via intermolecular interactions (Zhan et al., 2022). pH conditions exert a significant impact on all three stages.

First, during the diffusion stage, the pH-dependent protein particle size and solubility are key factors. Near the pI, the diffusion rate is significantly reduced due to the presence of large particle aggregates; in contrast, under pH conditions far from the pI, dissociated small-molecule proteins or oligomers exhibit higher diffusion coefficients and can rapidly occupy the interface (Shen, Dai, et al., 2023). Second, in the conformational rearrangement stage, pH-induced structural loosening enables protein molecules to unfold more easily at the interface, exposing internal hydrophobic amino acid residues and thus enhancing interfacial anchoring capacity (Tian et al., 2020). For example, studies have shown that rice protein treated with pH shifting exhibits a faster surface pressure rise rate at the air-water interface, indicating a significant acceleration of its adsorption kinetics (Shen, Dai, et al., 2023). Furthermore, the rheological properties of the interfacial film are also regulated by pH. Under suitable non-isoelectric pH conditions, adsorbed protein molecules can form a more compact network structure, cross-linked via disulfide bonds, hydrogen bonds, and hydrophobic interactions, thereby increasing the elastic modulus and viscous modulus of the interfacial film (Jin et al., 2024; Wang et al., 2020). This high-strength interfacial film can effectively inhibit the coalescence and Ostwald ripening of bubbles or droplets, thereby improving the long-term stability of foams or emulsions (Zhan et al., 2022).

#### 3.5.2 Ionic strength

Ionic strength, a key physicochemical parameter regulating protein conformation and interfacial adsorption behavior, exerts a significant and complex influence on the dynamic adsorption of rice protein at oil-water or air-water interfaces. The dynamic adsorption process of rice protein at interfaces typically follows a diffusion-controlled mechanism, which is subsequently limited by conformational rearrangement (Wang et al., 2026). Ionic strength modulates this process by affecting the diffusion coefficient and interfacial energy barrier. In the early stage of adsorption, increased ionic strength typically promotes the diffusion of rice protein toward the interface. This is because electrostatic screening reduces the energy barrier for protein molecules approaching the interface, allowing more molecules to overcome repulsive forces and adsorb onto the interface (Sarigiannidou et al., 2022; Tian et al., 2021). Studies have shown that at moderate ionic strength, the rate of interfacial pressure rise for rice protein accelerates, indicating improved adsorption kinetics (Abd Rahim et al., 2024). Furthermore, ion-induced treatments (e.g., combined with pH shifting) can further unfold the compact structure of rice protein, significantly enhancing its foaming capacity and foam stability—an effect attributed to the thickening of the interfacial adsorption layer and increased viscoelasticity (Yang, Chen, et al., 2024). However, the final equilibrium adsorption capacity depends on the packing density and conformational state of the protein at the interface. High ionic strength may lead to the formation of large aggregates in the bulk phase; although individual aggregates have greater mass, their specific surface area decreases, and steric hindrance effects may restrict tight packing at the interface, resulting in reduced saturated adsorption capacity (Abd Rahim et al., 2024; Han, Liu, & Tang, 2023). In contrast, the partially unfolded state of rice protein under moderate ionic strength allows molecules to form a denser, more uniform film at the interface, thereby achieving higher interfacial pressure and better emulsification stability (Ma et al., 2023; Tian et al., 2021).

#### 3.5.3 Protein concentration

Protein concentration is a key determinant of both the kinetics and equilibrium of interfacial adsorption. Within a certain concentration range, increasing the protein concentration enhances the diffusion rate to the interface, accelerates the reduction of interfacial tension, and shortens the time required to reach adsorption equilibrium (Ma et al., 2023; Rodríguez Patino et al., 2004). However, interfacial adsorption does not increase indefinitely. Beyond a certain threshold, adsorption may reach saturation, or protein molecules may begin to aggregate in the bulk phase rather than adsorb at the interface. This behavior is analogous to the critical micelle concentration (CMC) observed in surfactants, where concentrations above the CMC lead to micelle formation in the bulk solution rather than further interfacial adsorption (Corti & Degiorgio, 1977; Lebouachera et al., 2024). Although the self-assembly behavior of proteins differs from that of small-molecule surfactants, a similar threshold (CMC) exists for proteins, beyond which the adsorption rate and capacity at the oil-water interface stabilize, while excess protein tends to form aggregates in the bulk phase (Fig. 5) (Zhang et al., 2017). In addition, upon reaching the CMC, proteins form more complete and robust interfacial films, thereby significantly enhancing the stability of emulsions and foams (Qiao et al., 2020; Xiong et al., 2020; Zhang, You, et al., 2025). However, once the CMC is reached or exceeded, foaming and emulsifying performance may plateau or even slightly decline, likely due to increased solution viscosity caused by the formation of aggregates or micelle-like structures (Fan et al., 2024; Liu & Tang, 2014; Wei & Liu, 2000; Xiong et al., 2020).

**Fig. 5:**
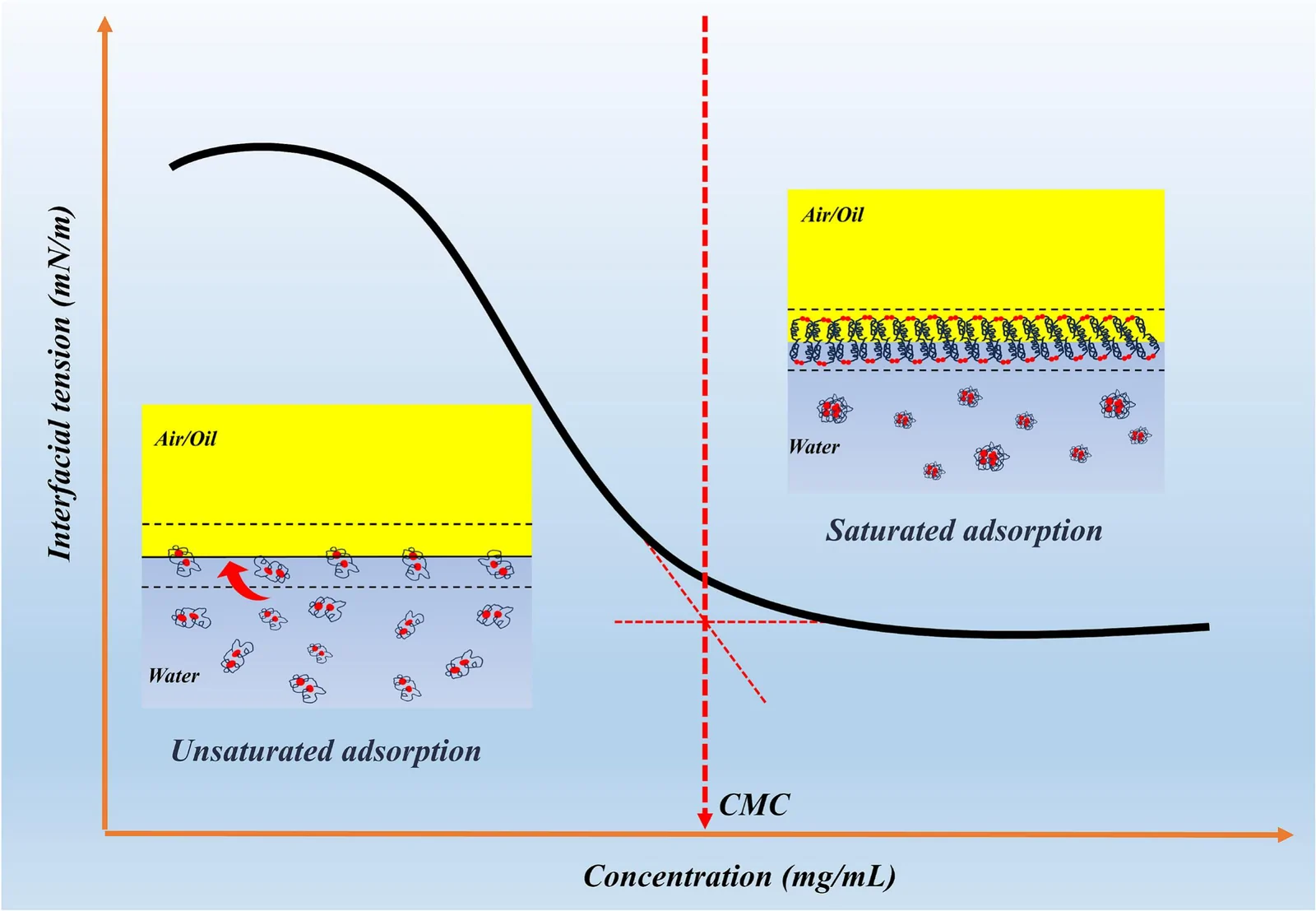
Determination of the critical micelle concentration of protein molecules by the surface tension method.

#### 3.5.4 Temperature

The influence of temperature on the dynamic interfacial adsorption behavior of proteins is a complex physicochemical process involving conformational changes, thermodynamic and kinetic equilibrium, and interfacial rheological properties. For rice protein, its dynamic adsorption at oil-water or air-water interfaces follows a diffusion-controlled mechanism, which is subsequently limited by conformational rearrangement and interfacial interactions (Kontogiorgos & Prakash, 2023). Temperature impacts this process primarily through two stages: the diffusion stage and the rearrangement stage.

In the diffusion stage, according to the stokes-einstein equation, increasing temperature reduces the viscosity of the continuous phase and enhances the thermal kinetic energy of protein molecules, which should theoretically accelerate diffusion (Sun, Roos, & Miao, 2024). However, for rice protein, the heat-induced increase in aggregate size offsets the benefits of reduced viscosity, leading to a non-monotonic change in the effective diffusion coefficient (Abd Rahim et al., 2024). In the rearrangement stage, moderate heating increases the flexibility of protein molecules, enabling them to unfold faster at the interface and form a dense interfacial film (Xiao et al., 2025). For example, our team previously found that heat treatment at 65 °C optimizes the interfacial properties of rice glutelin by promoting the transition from a rigid to a more flexible structure, thereby enhancing the viscoelasticity of the interfacial film (Wang et al., 2026). Furthermore, temperature treatment not only affects adsorption capacity but also profoundly alters the rheological properties of the interfacial film. Protein films formed via dynamic interfacial adsorption require a certain degree of viscoelasticity to resist droplet coalescence (Zhao et al., 2025). Rice protein films formed at the interface after heat treatment typically exhibit a higher interfacial elastic modulus, as unfolded protein molecules more readily form networks of intermolecular disulfide bonds and hydrophobic interactions (Lian et al., 2024; Luo, Wang, et al., 2023). This enhanced interfacial network structure improves the emulsion's resistance to thermodynamically unstable factors such as Ostwald ripening and gravitational sedimentation (Luo, Wang, et al., 2023). However, excessively high temperatures may cause excessive cross-linking of proteins in the bulk phase, forming large insoluble particles. While these particles can stabilize emulsions via the Pickering mechanism, their dynamic adsorption behavior differs from that of soluble proteins, characterized by irreversible particle adsorption rather than molecular-level unfolding (Son et al., 2025; Wen et al., 2024).

## 4 The relationship between the interfacial properties of rice protein and their foaming and emulsifying properties

The interfacial properties of rice protein, especially their emulsifying and foaming properties, are critical determinants of their practical applications in the food industry. These functional properties are not only intrinsically linked to the molecular structure of the protein, but are also substantially influenced by their adsorption behavior at air–water or oil–water interfaces, conformational changes upon interface binding, and the physicochemical characteristics of the resulting interfacial films (Patino et al., 1997; Sanchez & Patino, 2005; Tao et al., 2024; Zhang, Diao, et al., 2022).

### 4.1 The relationship between rice protein adsorption at the air–water interface and foaming performance

The adsorption behavior of rice protein molecules at the air-water interface exerts a decisive influence on their foaming properties, including foaming capacity and foam stability (Fig. 6). Therefore, a comprehensive understanding of the adsorption mechanisms of this protein at this interface is critical for their effective utilization as foaming agents in the food industry. This adsorption process typically consists of several sequential stages, each involving complex molecular events and kinetic regulation.

I. Diffusion and initial adsorption stage: In the initial stage, protein molecules rapidly migrate from the bulk solution to the air-water interface primarily via diffusion and convective transport mechanisms (Onaizi, 2018; Zhou et al., 2006). The rate of this process is influenced by factors such as protein concentration, molecular weight, and solution viscosity (Shen, Zheng, et al., 2023). For natural rice proteins, their relatively low solubility (e.g., the solubility of natural rice protein can be as low as 1.84%) severely limits their uniform dispersion in the aqueous phase and their ability to diffuse and migrate to the air-water interface (Gomes & Kurozawa, 2023; Wang et al., 2018; Wu et al., 2021), thereby significantly reducing their interfacial diffusion rate and initial adsorption efficiency.
II. Conformational changes and surface denaturation: Upon reaching the air-water interface, due to the substantial free energy difference between the interfacial environment and the bulk solution, protein molecules must undergo oriented spreading and partial unfolding—exposing hydrophobic side chains to the air phase while retaining hydrophilic groups in the aqueous phase—to minimize interfacial free energy (Han, Shen, et al., 2023). However, the extensive disulfide cross-linking network in rice glutelin endows it with extremely high structural rigidity, increasing the activation energy barrier required for conformational rearrangement (Li et al., 2023; Yang et al., 2023b). This rigid structure prevents rice proteins from completing sufficient unfolding and ordered rearrangement within the limited interface residence time, resulting in a loose, defect-prone adsorbed layer that cannot effectively resist bubble film drainage and rupture (Qi et al., 2023; Wu et al., 2023). In contrast, proteins with higher flexibility (e.g., whey protein or moderately denatured plant proteins) can spread more rapidly at the interface, forming a denser and mechanically stronger adsorbed layer (Chang et al., 2023; Sun, Xia, et al., 2024).
III. Formation of viscoelastic interfacial film and foam stability: As adsorption proceeds and conformational rearrangement deepens, a viscoelastic protein film gradually forms at the air-water interface—this is the core factor determining foam stability (Baldino et al., 2024; Kontogiorgos & Prakash, 2023). The viscoelastic characteristics of the interfacial film, particularly the interfacial dilatational modulus and shear modulus, directly reflect the film's ability to resist deformation and recover its original state (Chen et al., 2025; Wierenga et al., 2023). A high interfacial dilatational modulus can effectively inhibit bubble film drainage, weaken the Marangoni effect, and prevent film rupture, thereby significantly enhancing the foam's resistance to coalescence and Ostwald ripening (Sun, Roos, & Miao, 2024). However, the initial film formed by natural rice proteins is relatively loose, and its interfacial modulus is typically low (Wu et al., 2023). To enhance the mechanical strength of the natural rice protein interfacial film, a two-stage strategy (rapid adsorption-post reinforcement) can be employed: first, limit enzymatic hydrolysis or small-molecule complexes are used to increase the initial adsorption rate; subsequently, thermal induction, oxidation, or enzymatic transglutaminase reactions are applied to introduce intermolecular cross-linking (e.g., disulfide bond rearrangement or isopeptide bond formation) in the interfacial region, constructing a network structure with high viscoelasticity (Baldino et al., 2024; Lu et al., 2023; Wu et al., 2023). Additionally, complexes formed by rice protein with charged polysaccharides (e.g., gum arabic, carrageenan) or via Maillard reaction-induced glycosylation can further stabilize the interfacial film through electrostatic repulsion and steric hindrance effects, synergistically improving the long-term stability of the foam (Han et al., 2024; Li, Wan, et al., 2024; Zhang, Wang, Zhang, et al., 2024).

**Fig. 6:**
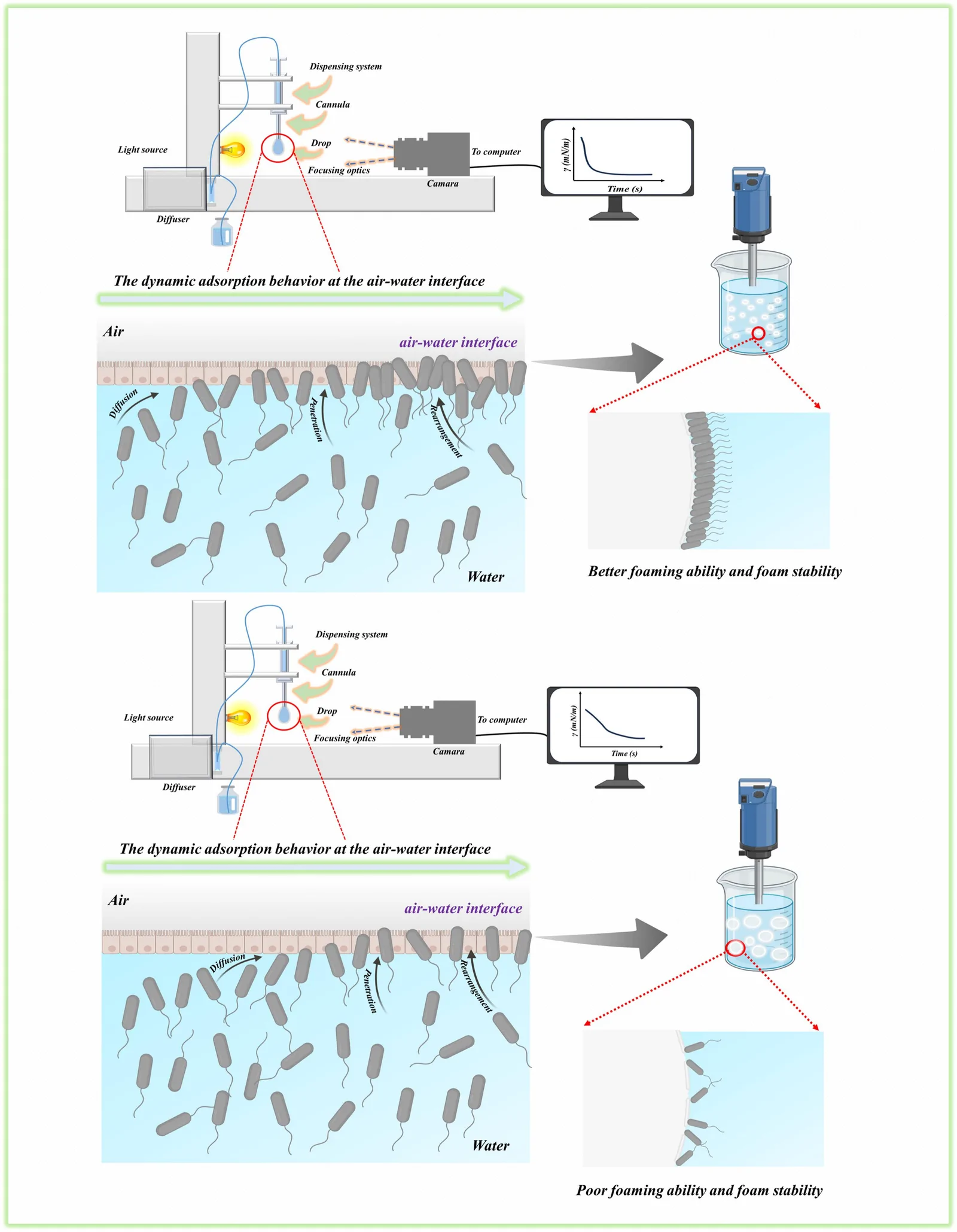
The relationship between the air - water interface adsorption of rice protein and its foaming property.

In conclusion, the adsorption behavior of rice protein at the air-water interface is a multi-stage process co-regulated by diffusion control, conformational rearrangement energy barriers, and the rheological properties of the interfacial film. The shortcomings in it foaming performance primarily stem from mass transfer limitations caused by low solubility and conformational rigidity induced by disulfide cross-linking.

### 4.2 The relationship between rice protein adsorption at the oil–water interface and emulsification performance

Rice protein, as a highly promising emulsifier in the plant-based food industry, its emulsifying activity index and emulsion stability largely depend on its dynamic adsorption behavior at the oil-water interface and the properties of the formed interfacial film (Fig. 7). Natural rice protein often exhibits low solubility and poor interfacial adsorption efficiency due to its compact globular structure and strong intermolecular interactions (Amagliani et al., 2017b). However, by understanding and regulating its diffusion, penetration, conformational rearrangement, and “confined denaturation” processes at the interface, its emulsifying properties can be significantly improved.

I. Diffusion stage: The initial step of protein emulsification is the diffusion of molecules from the bulk aqueous phase to the oil-water interface. For rice protein, the main bottleneck in this stage is that its low solubility in the natural state easily leads to intermolecular aggregation, forming large-sized aggregates, which significantly reduce the diffusion coefficient and interfacial adsorption rate (Li et al., 2023). The diffusion rate is influenced by multiple factors such as protein concentration, molecular weight, and solution viscosity, while the presence of aggregates further hinders the migration of effective monomers or small molecular oligomers to the interface (Igartúa et al., 2024).
II. Penetration stage: When protein molecules reach the interface, their hydrophobic segments are preferentially inserted into the oil phase, while the hydrophilic regions remain in the aqueous phase, a process accompanied by a significant reduction in interfacial tension. Interestingly, the adsorption rate of proteins at the oil-water interface is usually an order of magnitude faster than at the air-water interface, which is attributed to the stronger affinity of the oil phase for protein hydrophobic groups and the influence of dispersive interactions (Kontogiorgos & Prakash, 2023). For rice protein, the effective exposure of its internal hydrophobic groups is the key to determining penetration efficiency. However, most hydrophobic groups of natural rice protein are wrapped inside the molecule, limiting its initial interfacial penetration ability (Amagliani et al., 2017a). Modified technologies such as heat treatment or ultrasound-assisted alkaline treatment can induce partial unfolding of the protein, expose more hydrophobic residues and thus enhance its penetration ability in the oil phase (Li et al., 2016; Zhao et al., 2020).
III. Conformational change and rearrangement: At the oil-water interface, the adsorbed protein undergoes structural unfolding or conformational rearrangement to maximize the interfacial contact area and reduce the interfacial free energy. During this process, oil phase molecules can penetrate into the hydrophobic regions of the protein layer and partially solvate the hydrophobic residues, thereby limiting the excessive unfolding of the protein and forming a “confined denaturation” state (Zhan et al., 2022). This confined denaturation results in interfacial films formed by rice protein usually having a lower viscoelastic modulus but better flexibility and dynamic responsiveness, which helps resist coalescence caused by droplet collision (Wang, Ran, et al., 2023). In addition, the rearrangement process of rice protein at the interface is affected by its secondary structure, such as changes in the content of *β*-sheets and *α*-helices, which directly affect the mechanical strength of the interfacial film (Liu et al., 2022). By regulating processing conditions, such as ultrasonic treatment or enzymatic cross-linking, the conformational rearrangement of rice protein at the interface can be optimized to form a denser and more stable interfacial film (Igartúa et al., 2024; Zhu et al., 2025).

**Fig. 7:**
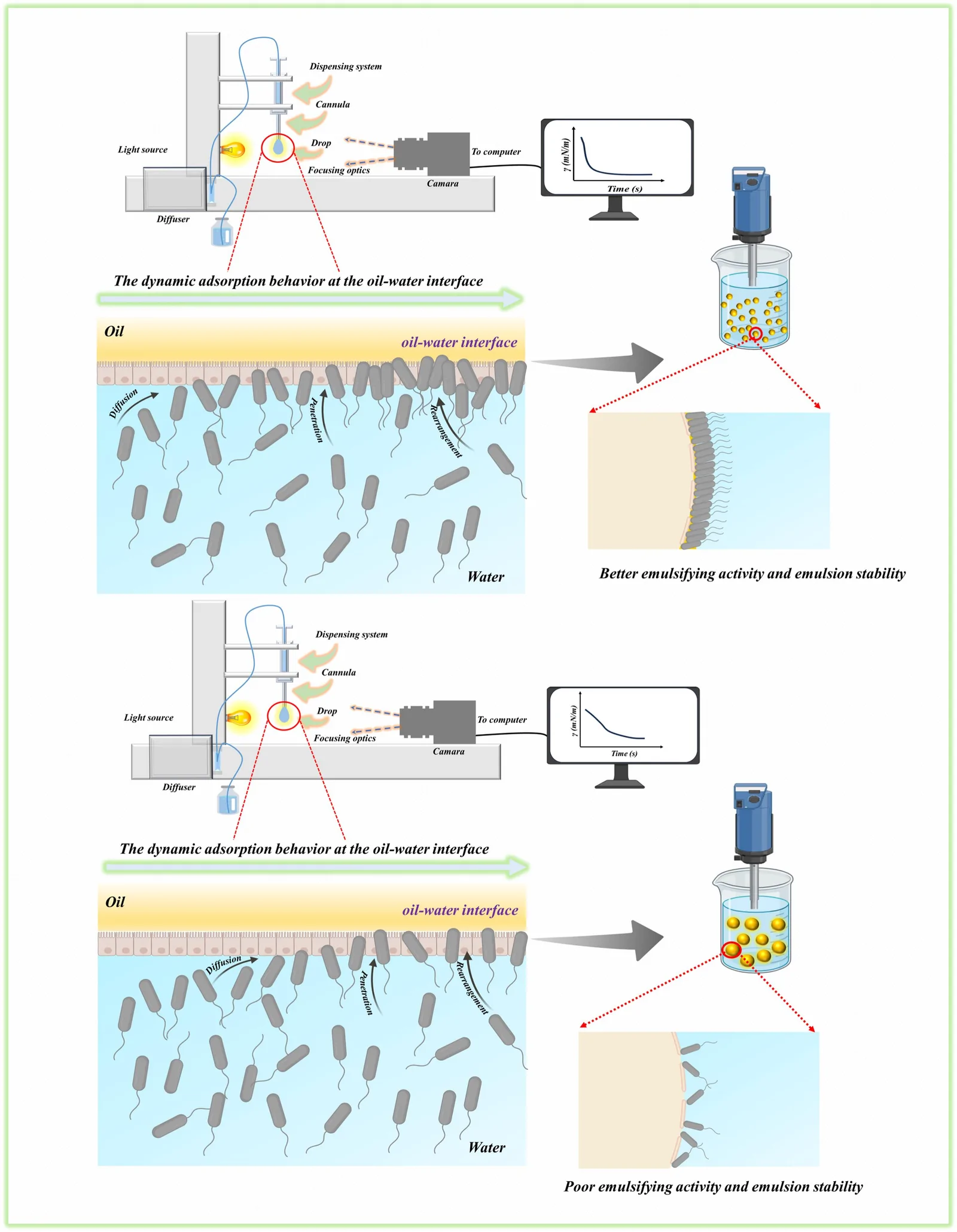
The relationship between the oil - water interface adsorption of rice protein and its emulsifying property.

In conclusion, the emulsifying properties of rice protein are closely related to its adsorption behavior at the oil-water interface. By deeply understanding the mechanisms of its diffusion, penetration, conformational rearrangement, and confined denaturation stages, and combining appropriate physical, chemical, or enzymatic modification methods, it is expected to overcome the structural limitations of natural rice protein and develop efficient and stable plant-based emulsification systems to meet the needs of the modern food industry for healthy and sustainable ingredients.

### 4.3 Differences in the interfacial adsorption mechanisms between protein-stabilized foams and emulsions

There exist significant differences in the interfacial adsorption behaviors of proteins at air-water and oil-water interfaces, as well as in their mechanisms contributing to the stability of foams and emulsions. These differences primarily stem from variations in the thermodynamic properties, intermolecular interactions, and interfacial rheological characteristics of the two interfaces. While rice protein, as amphiphilic macromolecules, can stabilize dispersed systems by reducing interfacial tension and forming viscoelastic films, their kinetic adsorption processes, conformational changes, and the mechanical properties of the final interfacial layers exhibit distinct interface-specific features (Mitropoulos et al., 2014).

I. Differences in interfacial thermodynamics and adsorption driving forces.
  The fundamental distinction between air-water and oil-water interfaces lies in the magnitude of interfacial tension and the nature of the dispersed phase. Air-water interfaces typically have higher interfacial tension (approximately 72 mN/m), whereas oil-water interfaces have lower interfacial tension (ranging from 20 to 50 mN/m, depending on oil polarity) (Sengupta & Damodaran, 1998). This high interfacial tension provides a stronger thermodynamic driving force for protein adsorption at air-water interfaces, leading to irreversible adsorption and dramatic structural rearrangement of protein molecules on bubble surfaces (Mitropoulos et al., 2014). In contrast, the presence of an oil phase introduces hydrophobic interactions and van der Waals forces, so protein adsorption at oil-water interfaces is regulated not only by electrostatic effects and steric hindrance but also by the properties of the oil phase (Rampon et al., 2004). Studies have shown that the adsorption rate of proteins at oil-water interfaces is typically an order of magnitude faster than at air-water interfaces, attributed to the stronger affinity of the oil phase for protein hydrophobic groups and the influence of dispersion interactions (Sengupta & Damodaran, 1998).
II. Differences in interfacial conformational changes and surface denaturation.
  The stability of proteins at interfaces largely depends on their post-adsorption conformational changes, i.e., the degree of surface denaturation. At air-water interfaces, due to the absence of solvation by oil-phase molecules, protein molecules tend to unfold to maximize the contact between hydrophobic residues and air, resulting in deeper surface denaturation and tighter intermolecular cross-linking (Mitropoulos et al., 2014; Walstra & De Roos, 1993). This extensive denaturation helps form solid-like interfacial films with high elastic moduli, effectively resisting bubble coalescence and liquid film drainage (Qiao et al., 2022). Conversely, at oil-water interfaces, oil-phase molecules can penetrate the hydrophobic regions of the protein layer and partially solvate hydrophobic residues, thereby limiting excessive unfolding of proteins (Rampon et al., 2004). This “confined denaturation” results in protein layers at oil-water interfaces typically having lower viscoelastic moduli but better flexibility and dynamic responsiveness (Liu et al., 2014).
III. Differences in interfacial rheology and stabilization mechanisms.
  Interfacial rheological properties are critical factors determining the stability of foams and emulsions. Foam stability mainly relies on the viscoelastic modulus of the interfacial film; a high elastic modulus can resist surface tension gradients during liquid film thinning, triggering the Marangoni effect to repair local weak points and effectively hinder drainage and coalescence between bubbles (Langevin, 2015; Maldonado-Valderrama & Patino, 2010; Wierenga et al., 2023). In contrast, emulsion stability is influenced not only by the mechanical strength of the interfacial film but also strongly depends on steric hindrance and electrostatic repulsion to prevent the approach and coalescence of oil droplets (Zhang, Fan, et al., 2022). At oil-water interfaces, the shear viscoelasticity of protein layers is usually lower than at air-water interfaces, but the thicker and softer interfacial layers formed can provide more effective steric hindrance (Wang, Wang, et al., 2023).

In summary, proteins stabilize foams at air-water interfaces primarily through strong surface denaturation and the formation of highly viscoelastic films, whereas at oil-water interfaces, they stabilize emulsions through moderate conformational adjustments, steric hindrance, and dynamic adsorption equilibrium. Understanding these mechanistic differences is crucial for optimizing the formulation of dispersed systems in food products.

## 5 Strategies for improving the interfacial properties of rice protein

As previously discussed, rice protein serves as a high-quality plant-derived protein source with notable advantages including low allergenic potential, environmental sustainability, and a well-balanced amino acid profile. These characteristics confer significant application potential in the food industry, particularly in the development of plant-based emulsifiers and foaming agents. Nevertheless, its limited interfacial properties hinder its widespread utilization as an effective plant-derived emulsifying and foaming ingredient. To address this restriction, various modification strategies have been proposed by researchers to improve the interfacial behavior of rice protein. These approaches primarily encompass physical modification, chemical modification, enzymatic modification, as well as hybrid strategies that integrate multiple modification techniques (Fig. 8).

**Fig. 8:**
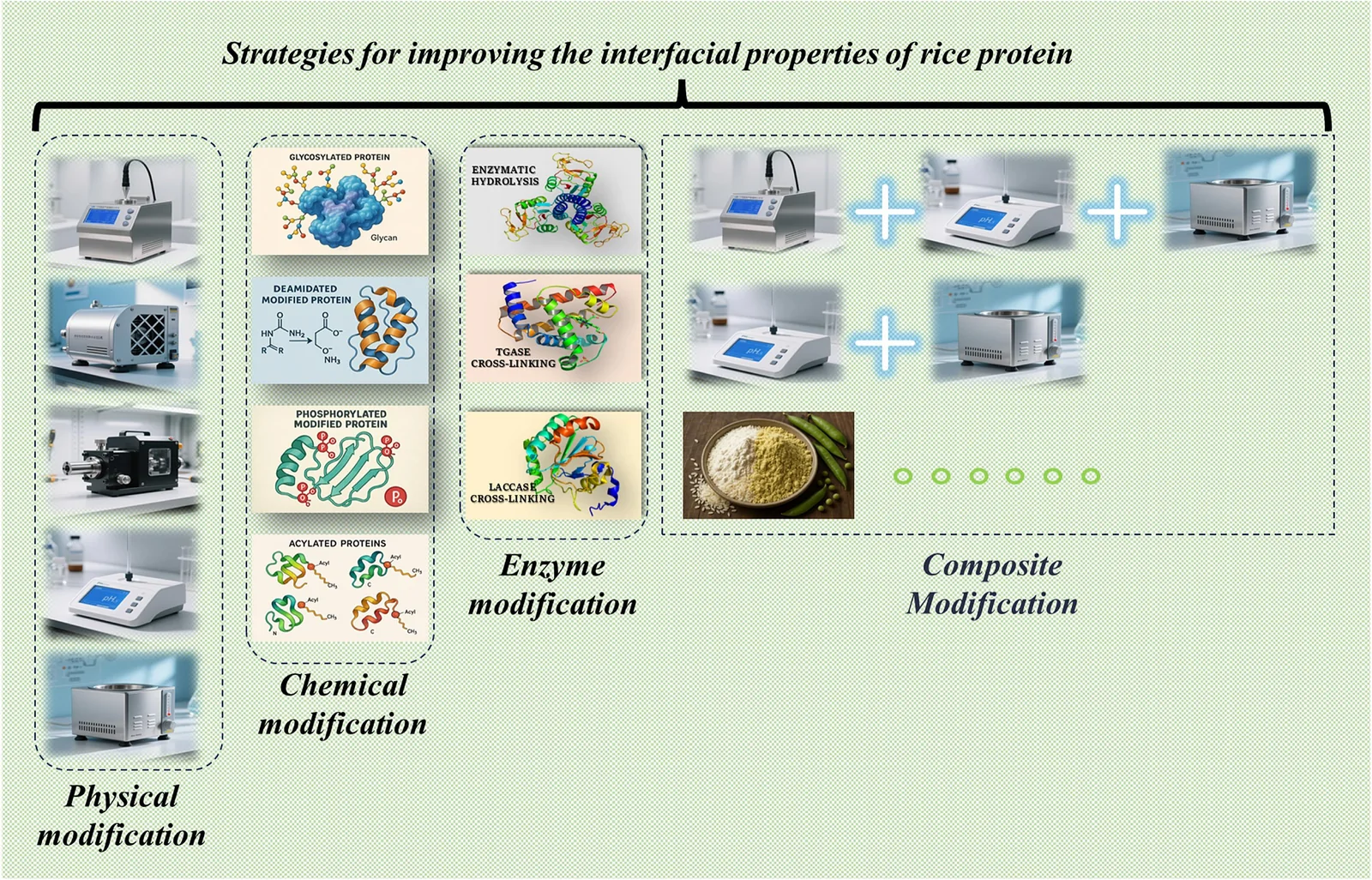
Main strategies for improving the interfacial adsorption behavior of rice protein.

### 5.1 Physical modification

Physical modification refers to the alteration of the spatial conformation and surface characteristics of rice protein through the application of mechanical forces, thermal treatment, or other physical interventions, with the aim of enhancing its interfacial activities. Commonly employed physical modification techniques include:

#### 5.1.1 Ultrasound

Ultrasonic technology, as a non-thermal processing method, has been extensively utilized in food science for the modification of proteins. This technique effectively enhances the interfacial adsorption behavior of rice protein at air–water or oil–water interfaces, thereby improving the stability of emulsions and foams. These improvements are primarily attributed to ultrasound-induced structural alterations in proteins, such as reduced particle size, increased solubility, exposure of hydrophobic groups, and enhanced molecular flexibility (Zhang, Zheng, et al., 2025). For example, in a study involving rice bran protein and epigallocatechin gallate complexes, moderate ultrasonic pretreatment at a power level of 425 W increased the trypsin sensitivity of adsorbed proteins from 3.20 to 3.73, while also elevating the surface hydrophobicity of unabsorbed proteins from 12.59 to 20.87. These observations suggest that ultrasound induces conformational changes in proteins at the interface, thereby enhancing their adsorption capacity and improving the rheological properties of the resulting emulsion (Li et al., 2025). Furthermore, research on the impact of ultrasonic-assisted treatment on rice bran protein–chlorogenic acid emulsions revealed that a power level of 400 W significantly reduced droplet size and improved size uniformity. Concurrently, emulsion viscosity decreased, while turbidity and interfacial protein content reached 24,758 and 9.34 mg/m^2^, respectively. These findings indicate that ultrasonic-assisted treatment substantially enhances emulsion stability (Wang, Wang, Li, et al., 2022). Similarly, in a study on the effects of ultrasound-assisted deamidation by protein glutaminase on the physicochemical properties of rice proteins, the results showed that this method significantly improved the foaming ability and emulsifying properties of rice proteins (Zhang, You, et al., 2025). In another study, the mixed solution of epigallocatechin gallate and rice bran protein was pretreated using gradually increasing ultrasonic power levels (200–500 W). This approach effectively modulated the absolute value of zeta potential, molecular flexibility, and surface hydrophobicity of the complex, thereby strengthening the interaction between the two components and further enhancing the stability of the emulsion system (Liu et al., 2024).

#### 5.1.2 High hydrostatic pressure

High hydrostatic pressure (HHP) has been demonstrated to substantially enhance the interfacial adsorption behavior of rice protein at both air–water or oil–water interfaces. This effect is primarily achieved through the application of uniform pressure to the liquid medium, typically within the range of 100–600 MPa, which facilitates the disruption and reorganization of non-covalent bonds within protein molecules—such as hydrogen bonds, hydrophobic interactions, and ionic bonds—leading to alterations in protein conformation and aggregation state (Wang, Wang, Yao, et al., 2022). In contrast to conventional thermal processing, HHP can induce protein unfolding or aggregation without significantly affecting covalent bonds, including peptide bonds, thereby exposing internal hydrophobic groups and sulfhydryl groups and enhancing interfacial activity (Liu et al., 2021; Rodiles-López et al., 2008). For example, studies have shown that HHP treatment can increase the solubility of rice bran protein by up to 76.69% (Yi & Liu, 2023). This improvement in solubility implies a greater availability of protein molecules for diffusion and adsorption at the interface, which is crucial for the formation of stable emulsions or foams (Shen, Dai, et al., 2023). Moreover, research has indicated that rice bran protein subjected to HHP pretreatment exhibits significantly improved emulsifying properties during subsequent enzymatic hydrolysis (Wang, Li, et al., 2025). This enhancement is attributed to the structural modifications induced by HHP, which render the proteins more susceptible to unfolding at the oil–water interface, thereby enabling more effective encapsulation of oil droplets and the formation of a stable emulsion system (Bai et al., 2021; Zhang, Zhang, Zhao, et al., 2024).

#### 5.1.3 Extrusion

Extrusion is a widely employed physical modification technique that utilizes high temperature, high pressure, and intense shear forces to induce protein denaturation, aggregation or depolymerization, thereby altering physicochemical and functional properties of proteins. In the case of rice protein, extrusion has been shown to significantly enhance emulsifying capacity and foaming performance. For example, following extrusion treatment, the solubility and emulsion stability of rice protein increased by 19.76% and 152.82%, respectively (Gao et al., 2022). Additional studies further support the beneficial effects of extrusion on the functional attributes of rice protein. One such study investigated the impact of extrusion processing on the functional properties and phytochemical composition of various rice bran varieties and found that the process markedly altered rice bran color, protein solubility, foaming capacity, as well as polyphenol and amino acid profiles (Kumar & Singh, 2024). Although this study mainly focused on rice bran, the mechanisms of protein structural changes and increased solubility revealed therein are similar to those in the research on the interfacial properties of rice proteins.

#### 5.1.4 pH shift

pH shift is an efficient and technically straightforward method for protein modification. It involves adjusting the pH of a protein solution to expose it to acidic or alkaline conditions before returning it to a neutral pH, thereby inducing structural reorganization of the protein and enhancing its functional properties. This approach promotes partial protein unfolding, exposes internal hydrophobic regions, and modifies surface charge distribution, which collectively improve the protein's adsorption capacity at interfaces and facilitate the formation of stable interfacial films. For example, research has demonstrated that pH-induced unfolding of rice protein isolates and its aggregates increase their susceptibility to subsequent modifications via ultrasound or thermal treatment. These combined treatments lead to more extensive protein unfolding, greater exposure of hydrophobic amino acids, and enhanced hydrolysis, ultimately resulting in oil–water emulsions with reduced droplet size and improved stability (Igartúa et al., 2024). In another study, rice protein isolate-stabilized emulsions prepared via alkaline pH shift treatment (pH 10) displayed smaller and more uniformly distributed droplets than those prepared under acidic conditions (pH 4), this improvement was attributed to the more balanced distribution of hydrophilic and hydrophobic amino acid residues in the protein structure under alkaline conditions, as well as the increased permeability and rearrangement rate of the interfacial film (Shen, Dai, et al., 2023).

#### 5.1.5 Heating

Thermal treatment serves as an effective strategy for enhancing the adsorption behavior of rice protein at both air–water or oil–water interfaces, thereby improving its emulsifying and foaming properties. For instance, research has demonstrated that moderate heating for 8 h significantly enhances the wettability, flexibility, diffusion rate, and adsorption capacity of rice bran protein fibril aggregates. These structural and functional modifications contribute to improved rheological properties and enhanced stability of high internal phase Pickering emulsions (Zhao et al., 2024). In another study, rice glutelin dissolved in an alkaline solution (pH 12) was subjected to heat treatment at varying temperatures (60, 80, and 100 °C) for durations of 30 and 60 min. Results indicated that heating at 100 °C for 60 min led to a threefold increase in solubility, along with a doubling of foaming capacity and significantly improved foam stability at pH 7 compared to the native protein. This enhancement was attributed to a reduction in the size of spherical glutelin aggregates (approximately 100 nm), a decrease in disulfide bond content, and a concurrent increase in surface net charge, surface hydrophobicity, and conformational flexibility (Zhao et al., 2020). Similarly, acidic thermal treatment has been employed to generate protein fibrils with enhanced interfacial adsorption capabilities. For example, glycosylated rice bran protein aggregates produced via acid-heat treatment (pH 2.0, 90 °C) have been shown to effectively stabilize oil–water emulsions (Luo et al., 2025).

### 5.2 Chemical modification

Chemical modification represents a key strategy for enhancing the functional properties of rice protein, primarily through structural modulation at the molecular level to influence its behavior at interfaces. Widely employed modification techniques encompass:

#### 5.2.1 Glycosylation

Glycosylation, as an effective chemical modification technique, has been extensively utilized to enhance the physicochemical properties and functional attributes of proteins. Its fundamental mechanism involves the covalent attachment of sugar molecules to protein structures, which modulates key parameters such as the hydrophilic/hydrophobic balance, molecular flexibility, surface charge, and spatial conformation. These structural alterations subsequently influence the adsorption kinetics of proteins at interfaces and their capacity to form stable interfacial films. For example, research has demonstrated that glycosylation significantly improves both the foaming capacity and foam stability of rice protein compared to its native form. This enhancement is primarily attributed to the incorporation of hydrophilic groups and the steric hindrance effect introduced by glycosylation, which collectively enhance the protein's adsorption efficiency and interfacial stability at the air–water interface (Cheng et al., 2021). Moreover, under macromolecular crowding conditions, the glycosylation of rice protein with dextran via the Maillard reaction has yielded stable glycosylated conjugates. These conjugates exhibited notable improvements in solubility, emulsifying properties, and foaming performance (Cheng et al., 2022).

#### 5.2.2 Deamidation

Deamidation, as a significant protein chemical modification technique, has been demonstrated to substantially enhance the interfacial adsorption behavior of rice protein, thereby improving its functional properties within food systems, particularly in terms of emulsion stabilization. This enhancement primarily stems from the ability of deamidation to modulate the structural and physicochemical characteristics of rice protein, including solubility, surface hydrophobicity, zeta potential, and molecular flexibility (Guan et al., 2017; Yang, Wu, et al., 2024; Zhang, You, et al., 2025). For instance, recent studies have revealed that rice protein subjected to deamidation using natural deep eutectic solvents exhibits markedly improved solubility and emulsifying properties (Shi, Xie, Ouyang, Shi, et al., 2024). Similarly, deamidated rice protein concentrates have been shown to adsorb more effectively at interfaces and form denser, more stable interfacial films on oil droplet surfaces, thereby significantly enhancing emulsion stability and emulsifying capacity (Vidotto et al., 2024). Furthermore, enzymatic deamidation enables precise regulation of surface properties while preserving the overall structural integrity of the protein, thus optimizing its interfacial adsorption performance (Luo, Deng, et al., 2023). For example, ultrasound-assisted glutaminase-mediated deamidation has been found to reduce the particle size of rice protein, increase the absolute value of zeta potential, and improve both foaming and emulsifying capabilities (Zhang, Zheng, et al., 2025). However, the extent of deamidation must be carefully controlled. Excessive deamidation may result in increased electrostatic repulsion between protein molecules, which can hinder rapid interfacial adsorption and ultimately weaken the mechanical strength of interfacial films, thereby compromising the stability of both emulsion and foam systems.

#### 5.2.3 Phosphorylation

Phosphorylation represents a valuable chemical modification strategy that can substantially enhance the adsorption behavior of rice protein at both air–water or oil–water interfaces, thereby significantly improving its functional properties, particularly in terms of emulsification and foaming capacity. This enhancement is primarily attributed to the regulatory effects of phosphorylation on protein structural flexibility, surface charge characteristics, and interfacial interaction dynamics (Hu, Zhou, Zeng, Du, et al., 2024; Hu, Zhou, Zeng, Xia, et al., 2024). For example, a study demonstrated that rice glutelin isolated from glutinous rice, when modified with varying concentrations (1%, 4%, and 7%) of sodium tripolyphosphate, exhibited reduced particle size and increased absolute zeta potential values following phosphorylation. Moreover, surface morphology analysis revealed the presence of distinct roughness features, including microscale depressions and pore-like structures. Most notably, phosphorylation significantly improved both the emulsifying and foaming properties of the protein (Wang et al., 2019). Despite the promising potential of phosphorylation in modulating the interfacial behavior of rice proteins, several limitations remain. Specifically, the underlying mechanisms governing the dynamic adsorption of proteins at interfaces, the kinetics of interfacial film formation, and the rheological properties of the resulting interfacial layers require further systematic investigation to fully elucidate their roles and optimize practical applications.

#### 5.2.4 Acylation

Acylation, a widely employed chemical modification technique, involves the introduction of acyl groups—such as acetyl and succinyl—into protein molecules, leading to substantial alterations in their molecular structure and physicochemical properties. These modifications subsequently influence the protein's adsorption behavior at interfaces. In the case of rice protein, acylation has been shown to enhance interfacial adsorption performance, thereby significantly improving its emulsifying and foaming capabilities within food systems. For example, research indicates that succinic anhydride induces structural changes in rice glutelin, as demonstrated by increased solubility, reduced particle size, and elevated absolute zeta potential values. These changes collectively contribute to enhanced emulsifying and foaming properties (Luo et al., 2024). Nevertheless, excessive acylation may result in over-unfolding or even polymerization of the protein structure. This not only weakens their functional properties but may also affect the digestion and absorption of proteins, and even trigger potential allergic risks (Ai et al., 2022; Luo et al., 2024). Furthermore, the scalability of acylated rice protein production and the associated cost-efficiency remain key challenges that must be addressed to facilitate its broader industrial application.

### 5.3 Enzyme modification

Enzymatic modification can affect the adsorption and spreading behavior of proteins at the two-phase interface by altering their molecular structure, molecular weight, hydrophilicity/hydrophobicity, and surface charge characteristics (Xiong et al., 2021; Zheng et al., 2024), thereby enhancing their performance in terms of emulsification and foaming. Specific modification methods include:

#### 5.3.1 Enzymatic hydrolysis

Enzymatic hydrolysis is a widely employed technique for protein modification, typically carried out through the catalytic activity of proteolytic enzymes such as papain, alkaline protease, and neutral protease. This process effectively reduces the molecular weight of rice protein, enhances its diffusion rate, and thereby facilitates faster adsorption at the oil–water interface (Almeida et al., 2019; Zheng et al., 2024). For example, rice protein subjected to papain-mediated hydrolysis combined with fibrillation treatment exhibits substantial alterations in its secondary and tertiary structural conformations, leading to improved solubility and enhanced emulsifying capacity (Qi, Luo, et al., 2024). In parallel investigations, rice bran protein pre-treated with high hydrostatic pressure followed by limited trypsin hydrolysis demonstrated a marked increase in both the emulsifying activity and emulsifying stability (Wang, Liu, et al., 2025).

#### 5.3.2 Transglutaminase cross-linking

Transglutaminase (TGase) is an enzyme that catalyzes the formation of isopeptide bonds within or between protein molecules through a reaction involving the γ-carboxamide group of glutamine residues and the ε-amino group of lysine residues, resulting in the establishment of covalent cross-links among protein structures (Tang et al., 2023; Wu et al., 2023). These cross-linking reactions lead to an increase in protein molecular weight and promote the formation of polymeric networks, thereby enhancing both structural stability and the capacity for network formation (Tang, Roos, & Miao, 2024; Wu et al., 2023). For example, continuous TGase-mediated cross-linking has been demonstrated to significantly improve the foaming capacity and foam volume ratio of rice bran protein while simultaneously reducing the rate of liquid drainage in the foam system (Wu et al., 2023). Similar research has also indicated that after hydrolyzing (using pepsin or papain), cross-linking (using TGase), and combined treatments (pepsin + TGase or papain + TGase) of a mixture of rice protein and pea protein, their solubility and surface hydrophobicity significantly increase, and their emulsifying stability, foaming ability, and foam stability are also significantly improved (Tang et al., 2023).

#### 5.3.3 Laccase cross-linking

Laccase is a polyphenol oxidase capable of catalyzing the oxidation of phenolic compounds and facilitating the cross-linking and polymerization of proteins. This enzymatic cross-linking approach has garnered significant attention due to its environmentally friendly and efficient nature in enhancing the functional properties of proteins (Chai et al., 2020). Compared with other cross-linking enzymes such as TGase, laccase offers the distinct advantages of not requiring additional cofactors and being effective under mild reaction conditions (Wu et al., 2023). For example, a study evaluating the crosslinking characteristics between rice proteins and ferulic acid, gallic acid, or tannic acid through covalent and non-covalent binding of laccase found that the covalent effect with a higher polyphenol binding equivalent exhibited higher emulsifying activity than the non-covalent effect (Shi, Xie, Ouyang, Wang, et al., 2024). Nevertheless, current research on the application of laccase-mediated cross-linking to improve the air–water interfacial adsorption and foaming properties of rice protein remains limited. Future investigations may focus on this underexplored area to further expand the functional applications of laccase in protein modification.

### 5.4 Composite modification

Compared to single modification approaches, composite modification strategies demonstrate superior efficacy in enhancing the interfacial adsorption performance of rice protein and its associated functional properties, including emulsification and foaming (Wang, Chen, et al., 2025; Wang, Ettelaie, & Sarkar, 2025; Wu et al., 2023). This enhanced performance is primarily attributed to the ability of composite modification to more comprehensively modulate the structural characteristics, solubility, flexibility, and surface activity of rice protein, thereby optimizing its adsorption behavior and film-forming capacity at the two-phase interface. For example, studies have shown that limited enzymatic hydrolysis of rice bran protein following high hydrostatic pressure pretreatment yields a hydrolysate with a degree of hydrolysis of 8%, which exhibits an improved molecular weight distribution, reduced particle size, and enhanced emulsifying properties (Wang, Hua, et al., 2025). Another investigation revealed that the combination of ultrasonic treatment and alkaline heat treatment can significantly reduce the particle size of rice protein to approximately 129.67 nm while increasing the proportion of random coil structures. This structural change enhances protein flexibility and accelerates its diffusion and spreading at the oil–water interface, resulting in the formation of a more stable interfacial film and improved emulsification performance (Zhu et al., 2025). Further research has demonstrated that high-temperature heat treatment of rice glutelin dissolved in an alkaline solution (pH 12) disrupts intramolecular disulfide bonds and induces structural alterations at the primary, secondary, and tertiary levels. These modifications lead to a reduction in aggregate size, an increase in net surface charge, and improved structural flexibility. Consequently, the solubility of rice glutelin at pH 7 increases by approximately threefold, its foaming capacity doubles, and foam stability is significantly enhanced (Zhao et al., 2020). In addition, studies on rice bran protein using pH-shifting combined with the addition of ions such as Na^+^ and Ca^2+^ have shown improvements in foaming properties and foam stability. These enhancements are primarily due to reductions in particle size and increased conformational flexibility, which promote greater adsorption capacity at the air–water interface and foam stabilization (Yang, Chen, et al., 2024). Other studies have indicated that the presence of polyphenols can alter interfacial wettability and surface tension, promoting the formation of rice protein–polyphenol complexes. The assembly behavior of these complexes at the oil–water interface plays a crucial role in determining emulsion stability (Chen et al., 2023; Kim et al., 2024). Moreover, the formation of complexes between rice protein (or its hydrolysates) and polysaccharides at the oil–water interface has also been shown to significantly influence emulsifying properties (Chen et al., 2023). A further study found that the synergistic combination of pea protein and rice bran protein can enhance emulsion stability. This effect is attributed to the increased viscosity of the continuous phase provided by soy protein and the reduced interfacial tension achieved by rice bran protein (Tong et al., 2024).

Overall, physical modification primarily regulates the molecular structure of rice protein (e.g., reducing particle size, enhancing solubility, exposing hydrophobic groups, and increasing molecular flexibility), which significantly improves its adsorption kinetics and coverage at oil–water or air–water interfaces, thereby effectively enhancing emulsifying and foaming properties. Chemical modification, on the other hand, systematically adjusts the hydrophilic–hydrophobic balance, molecular flexibility, surface charge distribution, and spatial conformation of rice protein through covalent modifications (e.g., glycosylation, phosphorylation, acylation) or amino acid side-chain modifications (e.g., deamidation), thereby optimizing its interfacial adsorption behavior and interfacial film stability. Enzymatic hydrolysis uses proteases to directionally degrade rice protein into low-molecular-weight peptides, which can simultaneously reduce molecular weight, accelerate interfacial diffusion rate, and improve aqueous solubility; transglutaminase and laccase, however, catalyze intra- or intermolecular cross-linking reactions to form protein polymer networks with higher mechanical strength and thermal stability, strengthening the structural integrity of the interfacial film. In addition, combined modification strategies often offer advantages over single methods, as they can synergistically regulate the multi-dimensional structural properties of rice protein (e.g., conformational flexibility, dissolution behavior, surface activity, and aggregation tendency), thereby more comprehensively optimizing its interfacial adsorption efficiency and film-forming ability.

However, beyond modification methods and process parameters, the effectiveness of regulating the emulsifying and foaming properties of rice protein may also be influenced by rice variety and raw material source (as described in Section 2.1). Rice variety (e.g., indica, japonica) and raw material source (e.g., polished rice, rice bran) are also key variables affecting the interfacial functionality of rice protein—rice proteins from different sources exhibit significant differences in the proportion of major components (e.g., relative abundance of glutelin, globulin, albumin, and prolamin), initial aggregation state, and secondary structural characteristics, which directly limits the reproducibility and comparability of modification effects. Notably, most current studies focus on modification strategies themselves (e.g., extraction optimization, physical/chemical/enzymatic treatments) and lack sufficient reporting on the variety information and raw material sources of the rice protein used, which seriously undermines the comparability of cross-study results and the reliability of mechanism analysis.

### 5.5 Industrial feasibility, scalability, and regulatory considerations of modification strategies

While the various modification strategies mentioned above show great potential in enhancing the emulsifying and foaming properties of rice protein, their transition from laboratory research to industrial application still requires comprehensive consideration of industrial feasibility, scalability, and potential regulatory issues.

#### 5.5.1 Challenges in industrial feasibility and scalability

While physical modification methods (e.g., ultrasonic treatment and high hydrostatic pressure technology) can regulate emulsifying and foaming properties by altering protein structure and optimizing their adsorption capacity at oil-water or air-water interfaces—thereby creating possibilities for industrial applications in the food sector—high equipment costs and technical limitations in large-scale production (such as limited penetration depth of ultrasound or product size restrictions for high hydrostatic pressure) remain key barriers to their large-scale implementation (Lin et al., 2020). Although extrusion technology offers advantages of continuous production and cost-effectiveness, its high energy consumption and limitations to low-moisture products also need to be considered (Zheng, Regenstein, et al., 2023). For enzymatic modification, the relatively high cost and greater difficulty in precisely controlling enzymatic reactions (e.g., degree of hydrolysis) at an industrial scale may lead to reduced product flavor stability (e.g., formation of bitter peptides) or functional stability (Li, Zhou, et al., 2024). Chemical modification methods such as phosphorylation and acylation require the use of chemical reagents, and the recovery of reagents, purification of products, and waste disposal increase industrial costs and environmental burdens (Yang et al., 2023a). Although composite modification strategies can produce synergistic effects, their process flows are usually more complex, involving optimization of multiple steps and parameters; maintaining the stability and consistency of such complex processes to ensure uniform product quality is a major challenge in industrial production (Sun et al., 2025). While pH regulation is simple to operate, precise pH control to avoid over-reaction and ensure product functionality is difficult to achieve in large-scale reactors (Zheng et al., 2024).

In addition, most current studies evaluate the performance of modified rice protein only in simple model systems; however, in actual food systems such as baked goods, dairy products, and plant-based meat, rice protein interacts with multiple components including fats, sugars, salts, other proteins, and polysaccharides (Lu et al., 2024). These complex interactions may significantly affect its interfacial adsorption behavior, emulsifying and foaming properties, and even lead to performance degradation (Zhang et al., 2023). Therefore, more research on practical applications in complex food matrices is urgently needed.

#### 5.5.2 Regulatory and safety considerations

In addition to facing challenges related to industrial feasibility and scalability, these modification strategies must also address multiple considerations at the regulatory and safety levels. From the perspective of nutritional value and biosafety, core concerns focus on three aspects: first, potential nutritional loss—some modification methods may affect the nutritional quality and digestive properties of rice protein. For example, glycosylation may reduce protein nutritional value (Xu et al., 2023), while deamidation and acylation may alter protein digestibility (Chen et al., 2021; Vidotto et al., 2024; Yang, Wu, et al., 2024). Therefore, in practical applications, comprehensive evaluations of amino acid scores, digestibility, protein efficiency ratios, and bioactivity are required to ensure nutritional value remains uncompromised and meets food safety standards. Second, allergenicity risks—although rice protein has lower allergenicity compared to common allergens like wheat and soy, this does not mean modified products are absolutely safe. Chemical or enzymatic modification may alter the protein's spatial conformation, exposing linear epitopes originally hidden inside the molecule, or form new conformational epitopes through cross-linking and glycosylation, potentially enhancing allergenicity (Liu et al., 2023; Zhou et al., 2022). Thus, strict allergenicity assessments are needed for verification. Third, safety of residues and by-products—reagents used in chemical modification and their by-products pose another critical safety risk. For instance, reagents like acid anhydrides or acyl chlorides used in acylation reactions, if not thoroughly cleaned, may remain in the final product and have potential cytotoxicity or irritancy (Abedi & Pourmohammadi, 2021). While enzymatic modification is generally considered milder, evaluations of enzyme source, purity, and potential endotoxins or contaminating proteins are still necessary (Zhang, You, et al., 2025), and their toxicological safety requires systematic assessment and regulatory approval.

At the regulatory level, modified rice protein, as a food ingredient or novel food component, must meet strict compliance requirements. Taking the European Union as an example, if modification causes significant changes to protein structure, it may be classified as a novel food, requiring comprehensive safety assessment by the European Food Safety Authority, including toxicology testing, exposure assessment, and allergenicity analysis (Garciarena et al., 2025; Laganaro et al., 2025). In the United States, it may need to go through generally recognized as safe designation or food additive approval procedures (Kedar et al., 2024). In China, it must follow management requirements for novel food ingredients or food additives, submitting a complete safety assessment package covering production process specifications, component purity standards, residue limits, and labeling regulations (Tang, Yao, et al., 2024; Zheng et al., 2024). In summary, regulatory authorities worldwide are gradually adopting modern methodologies to enhance assessment efficiency, while the core requirement remains unchanged: ensuring the safety of modified proteins throughout their entire life cycle (Cattaneo et al., 2023).

## 6 The application potential of modified rice protein in the food industry

As previously discussed, rice protein exhibits significantly enhanced emulsifying and foaming properties following various modification treatments. These improvements establish a strong scientific basis for its utilization as a plant-derived emulsifier and foaming agent in food industry applications (Fig. 9). The primary application potentials of modified rice protein in the food sector are outlined as follows:

**Fig. 9:**
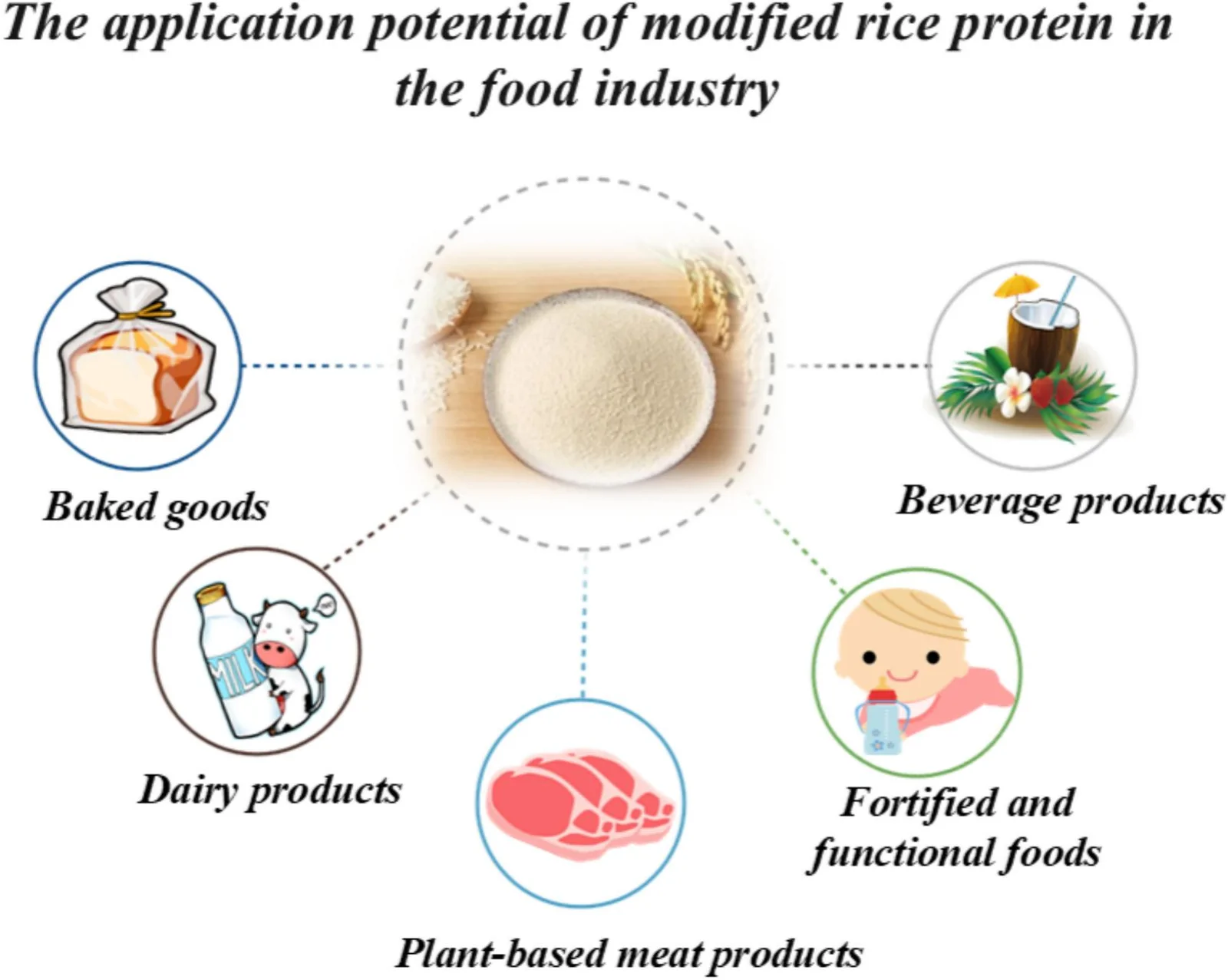
The application potential of modified rice protein as a plant-derived emulsifying and foaming agent in the food industry.

### 6.1 Baked goods

In the processing of baked products such as bread and cakes, dough or batter serves as the base matrix, forming a complex multiphase system composed of aqueous, oil, gas, and solid phases. Therefore, maintaining the processing stability of this system is crucial for optimizing the final quality of the products. Although natural rice protein exhibits certain interfacial activity, its native globular structure and highly ordered secondary structure make it prone to aggregation and precipitation in aqueous solutions, limiting its functional properties (Yang et al., 2023b). When applied to the complex multiphase system of baking dough or batter, natural rice protein typically fails to dissolve sufficiently and distribute uniformly, thus unable to effectively adsorb onto oil droplet surfaces to form a stable emulsified layer (Abd Rahim et al., 2024). The lack of emulsification stability leads to phase separation and aggregation of oil during mixing and baking, disrupting the uniformity of the dough's microstructure and ultimately affecting the texture and water retention of baked products (Pycarelle & Delcour, 2021). Additionally, natural rice protein has a slow adsorption rate at the air-liquid interface, and the formed interfacial film has low mechanical strength and lacks elasticity, making it difficult to effectively trap carbon dioxide gas produced during fermentation (Zhao et al., 2020). This directly results in insufficient expansion of gluten-free bread or cakes during baking, with rough and uneven internal pore structures, small product volume, hard texture, and short shelf life (Xu et al., 2020). In contrast, modifying rice protein through methods such as enzymatic hydrolysis, heat treatment, ultrasound-assisted processing, or chemical modification can significantly improve its solubility and interfacial activity, enabling it to diffuse to interfaces more rapidly and form robust viscoelastic interfacial films through intermolecular interactions (Igartúa et al., 2024; Xie et al., 2023; Yang et al., 2023a). Such interfacial films not only effectively prevent oil droplet coalescence and Ostwald ripening to maintain the thermodynamic stability of emulsions but also form a tough protective layer around bubbles to prevent their merging and rupture (Wang, Liu, et al., 2025; Zheng, Wei, et al., 2023). Especially in gluten-free baking systems, where the gas-retaining capacity provided by gluten networks is absent, the stable foam structure formed by modified rice protein is crucial for maintaining batter volume and forming a fine honeycomb-like texture (Zhao et al., 2022). Studies have shown that rice protein fibers hydrolyzed by proteases exhibit excellent foaming properties and foam stability in angel cake applications, significantly increasing the specific volume and softness of the cakes (Qi, Li, et al., 2024). In summary, while unmodified rice protein has significant limitations in baking applications due to low solubility and weak interfacial activity, appropriate modification techniques can significantly enhance its emulsifying and foaming properties. Modified rice protein can effectively maintain emulsion and foam structures in gluten-free baking systems by forming stable interfacial films, thereby improving product volume, texture, and sensory quality, and showing great potential to replace traditional synthetic emulsifiers and foaming agents.

### 6.2 Dairy products

With the increasing consumer demand for plant-based foods, rice protein demonstrates significant potential for application in the development of dual-protein dairy products, where it can partially substitute animal-derived proteins with plant-based alternatives. This approach holds considerable practical value for regions facing limitations in dairy supply or inadequate protein consumption (Hu et al., 2022). Furthermore, rice protein can also serve as an ideal non - dairy alternative to yogurt for lactose - intolerant individuals, providing them with favorable nutritional value and health benefits (Abdelshafy et al., 2024). However, the direct application of natural rice protein in dual-protein dairy products or plant-based yogurt faces multiple technical challenges. First, the solubility of natural rice protein is typically below 10% under neutral pH conditions, which prevents it from effectively adsorbing at the oil-water interface in emulsion systems and forming a stable interfacial film (Yang et al., 2023b). Direct addition of natural rice protein to dual-protein yogurt systems may result in rough texture, graininess, and severe whey separation and phase separation during storage. Second, natural rice protein has weak gelling ability and cannot form a fine, uniform three-dimensional network structure like casein or whey protein, leading to significantly lower viscosity and water-holding capacity in the final product compared to traditional dairy products or modified plant protein products (Roy et al., 2023). Additionally, the undesirable flavor of natural rice protein, such as astringency, is more pronounced without modification, severely affecting consumer sensory acceptance (Roy et al., 2025). In contrast, rice protein modified through various technical methods overcomes these limitations. Among them, enzymatic hydrolysis modification is one of the most effective methods to enhance the functional properties of rice protein. For example, hydrolyzed rice glutelin, as a plant-based emulsifier, can significantly reduce the surface tension in coconut milk emulsions and enhance the emulsion's resistance to processing stresses (e.g., heating, shearing), thereby effectively reducing phase separation and stratification (Phakthawat et al., 2023). Furthermore, in dual-protein emulsion systems, rice protein hydrolysates that partially replace whey protein not only maintain better physical stability but also improve in vitro digestibility, showing a more superior nutrient release pattern (Wang, Chen, et al., 2025). In addition to enzymatic hydrolysis, physical modification techniques such as high-pressure homogenization, ultrasonic treatment, and microwave-assisted enzymatic hydrolysis are also widely used. For instance, high-pressure homogenization combined with pH shifting treatment can significantly alter the secondary and tertiary structures of rice protein, increase its surface charge and flexibility, thereby greatly improving its emulsification stability and foam stability (Igartúa et al., 2024; Yang, Wu, et al., 2024). Moreover, chemical modification such as deamidation (using natural deep eutectic solvents based on citric acid) can effectively break hydrogen bonds inside the protein, improve its hydrophilicity, and thus enhance its application potential in dairy processing (Shi, Xie, Ouyang, Shi, et al., 2024). In summary, unmodified rice protein has significant limitations in dairy applications due to low solubility and poor functional properties. However, through modification methods such as enzymatic hydrolysis, physical field assistance, or chemical modification, the solubility, emulsifying properties, and gelling properties of rice protein can be significantly improved, making it a high-quality functional ingredient in dual-protein dairy products and plant-based yogurt.

### 6.3 Plant-based meat products

Plant-based meat, also known as plant-based meat substitutes or meat analogs, is developed to simulate the sensory and functional properties of traditional animal meat, including taste, flavor, appearance, and texture (Imran & Liyan, 2023). Modified rice protein has been used to construct protein-based food systems with meat-like textures, thereby effectively replicating the structural and sensory characteristics of animal-derived meat products (Rahaman et al., 2025). For example, studies have shown that rice protein can effectively replace soy protein in meat analog production via low-moisture extrusion, exerting significant impacts on parameters such as mechanical energy, expansion rate, bulk density, and textural properties (Lee et al., 2022). Additionally, rice protein isolate at its glass transition temperature has been used to regulate the formation of protein network density and fibrous structure in high-moisture soy protein-based meat analogs, helping to enhance their structural and textural similarity to traditional meat (Choi et al., 2026). However, the latest scientific evidence indicates that compared to modified rice protein, unmodified rice protein has significant limitations in constructing high-fidelity fibrous structures due to its inherent high hydrophobicity, low solubility, and lack of ability to form a continuous elastic network (Roy et al., 2025; Zheng et al., 2024), It typically exhibits low water-holding capacity and poor heat-induced gelation properties, leading to texturized plant proteins formed during low-moisture extrusion that are often overly hard and lack the juiciness after chewing (Webb et al., 2023; Zheng et al., 2024). Furthermore, due to insufficient disulfide bond exchange and hydrogen bond rearrangement capabilities, unmodified rice protein struggles to form layered fibrous structures similar to soy protein or wheat gluten during low-moisture extrusion; its extrudates usually present as granular or amorphous structures rather than the desired muscle fiber bundles (Patuk et al., 2026). On the other hand, while the flavor profile of unmodified rice protein is relatively neutral, its rough mouthfeel and grittiness limit its application in high-end plant-based meat products, especially in scenarios requiring simulation of the delicate texture of whole-cut meat (Roy et al., 2025). In summary, the application of unmodified rice protein in plant-based meat products is limited by its inherent physicochemical properties, making it difficult to independently construct high-quality meat-like fibrous structures. In contrast, modified rice protein improves the texture, water-holding capacity, and chewiness of the final plant-based meat products by enhancing its structure-directing ability and network-reinforcing effect during extrusion processing.

### 6.4 Fortified and functional foods

Rice protein, as a high-quality plant-based protein source, exhibits great application potential in infant formula, vegetarian diets, and gluten-free diets due to its relatively balanced amino acid composition, high biological value, and extremely low allergenicity (Roy et al., 2025; Yang et al., 2023a). While modification techniques (such as enzymatic hydrolysis, chemical modification, and physical treatment) can significantly improve the solubility and emulsification stability of rice protein, thereby meeting the requirements of high-end functional foods like infant formula emulsions and highly stable emulsions (Shi, Xie, Ouyang, Wang, et al., 2024; Wang, Yang, Rao, et al., 2022). Natural rice protein still has irreplaceable advantages in specific food fortification and basic functional applications. For example, natural rice protein retains its original amino acid profile; although lysine, as the first limiting amino acid, has a relatively low content, its sulfur-containing amino acids are sufficient, and its overall biological value is leading among plant proteins (Jayaprakash et al., 2022; Roy et al., 2025). For dietary fortification in the general population, natural rice protein, with its low allergenicity and gluten-free properties, becomes an ideal alternative to wheat protein (Roy et al., 2025). However, the solubility of natural rice protein under neutral pH conditions is extremely poor, mainly due to its compact globular structure and strong intermolecular hydrophobic interactions (Yang et al., 2023b). This low solubility directly limits its application in liquid food systems, making it difficult to form a stable colloidal dispersion system without modification. To overcome this restriction, modification technology has become a key solution. For instance, after modification treatments such as enzymatic hydrolysis or pH shifting, the peptide chains of rice protein unfold, exposing more hydrophilic groups, and the solubility is significantly improved, allowing for more effective digestion and absorption by the human body and the release of bioactive peptides (Chen et al., 2021; Singh et al., 2021). Additionally, covalent or non-covalent complexes formed by rice protein hydrolysates and phenolic acids like ferulic acid not only enhance the physical stability of emulsions but also endow the system with significant antioxidant activity, which can effectively protect unsaturated fatty acids in emulsions from oxidative damage—a function that natural proteins cannot achieve (Deng et al., 2024; Wang, Wang, Li, et al., 2022). In summary, the application strategy of rice protein needs to be precisely selected based on the requirements of the target product. In high-end food systems pursuing extreme solubility, emulsification stability, and additional antioxidant functions, modified rice protein processed via enzymatic hydrolysis, pH shifting, or complexation with phenolic acids demonstrates excellent performance. In the field of basic dietary fortification that focuses on complete nutrient retention, low allergenicity, and gluten-free labeling, natural rice protein continues to play an indispensable role relying on its inherent nutritional advantages and safety.

### 6.5 Beverage products

In coffee creamer, cocoa beverages, and other oil-containing drinks, a stable oil-water interface film is crucial for preventing fat floating and droplet coalescence (Akkaya Öner, 2025). However, natural rice protein exhibits slow diffusion at the oil-water interface and forms an interface film with low mechanical strength, making it difficult to maintain emulsion stability at low addition levels (Chen et al., 2023). In contrast, certain modification methods—such as limited enzymatic hydrolysis—can effectively reduce the molecular weight of the protein. When further combined with pH-shift treatment, these methods significantly expose hydrophilic groups, disrupt hydrophobic aggregation domains, and greatly enhance the protein's dispersibility and solubility, thereby improving its adsorption efficiency at the oil-water interface and promoting the formation and stability of a highly viscoelastic interface film (Igartúa et al., 2024; Nisov et al., 2020). Additionally, beverage processing typically involves pasteurization or ultra-high-temperature instant sterilization. Natural rice protein is prone to irreversible aggregation upon heating, leading to rough mouthfeel and precipitation (Igartúa et al., 2024). Through glycosylation or limited enzymatic hydrolysis modification, the thermal aggregation threshold of the protein can be lowered, and its solubility and dispersion stability after heat treatment can be improved (Nisov et al., 2020). Meanwhile, in functional beverages fortified with minerals like calcium and magnesium, divalent cations often induce protein precipitation. Modified rice protein, by introducing negatively charged groups or forming complexes with polysaccharides, can effectively shield against ion-induced aggregation, ensuring the stability of beverage systems with high mineral concentrations (Roy et al., 2025; Wei et al., 2024). This is of great significance for developing high-nutrient-density beverages targeting athletes or individuals with specific health needs. On another note, glycosylation modification, by introducing polysaccharide chains (e.g., arabinogalactan or guar gum derivatives), utilizes steric hindrance to prevent intermolecular aggregation of rice protein, further enhancing solution transparency and long-term storage stability (Yang et al., 2023a). This modified protein system is particularly suitable for acidic beverage environments with a pH of 3.0–4.0, holding promise for solving the technical challenge of flocculation in traditional plant proteins under acidic conditions (Shen, Dai, et al., 2023).

In summary, compared to natural rice proteins, modified rice proteins exhibit significant improvements in emulsifying and foaming properties, effectively overcoming the inherent limitations of natural rice proteins—such as low solubility, insufficient interfacial activity, and high molecular structural rigidity. As a result, they can play critical functional roles in various complex food matrices, including baked goods, dairy products, plant-based meat products, fortified and functional foods, and beverage systems. However, most current studies focus primarily on the modification techniques themselves (e.g., optimization of extraction processes and physical, chemical, or enzymatic modification), with insufficient systematic description and standardized reporting of the rice protein varieties (e.g., indica, japonica, or glutinous rice) and raw material sources (e.g., polished rice, rice bran, or brown rice) used. This severely undermines the comparability of results across studies and the scientific rigor and generalizability of mechanism analysis. In fact, beyond modification methods and their process parameters, the genetic background of rice varieties and compositional differences in raw material sources are also key variables influencing modification outcomes (as discussed in Section 2.1). These factors can significantly regulate the intrinsic structural properties of rice proteins, including hydrophilic-hydrophobic balance, surface net charge density, and conformational flexibility. In turn, this profoundly affects their adsorption kinetic behavior and interfacial film formation capacity at air-water or oil-water interfaces, ultimately leading to heterogeneity in the functional performance of modified rice proteins across different food application scenarios.

## 7 Future frontier strategies for improving the interfacial adsorption of rice protein

Although current technologies have demonstrated the capacity to enhance the interfacial adsorption behavior of rice protein as well as its emulsifying and foaming properties, this remains a multidisciplinary and multifaceted research endeavor. The core lies in overcoming the functional limitations of native rice protein through precise structural modifications and ingenious system construction, so as to enable it to play a greater role in the food field. Looking ahead, future strategies for improving the interfacial adsorption of rice protein may encompass approaches such as biological breeding, computational biology combined with molecular dynamics simulations, the establishment of protein structure–function databases integrated with machine learning algorithms for property prediction, and the development of rice protein-based smart materials (Fig. 10).

**Fig. 10:**
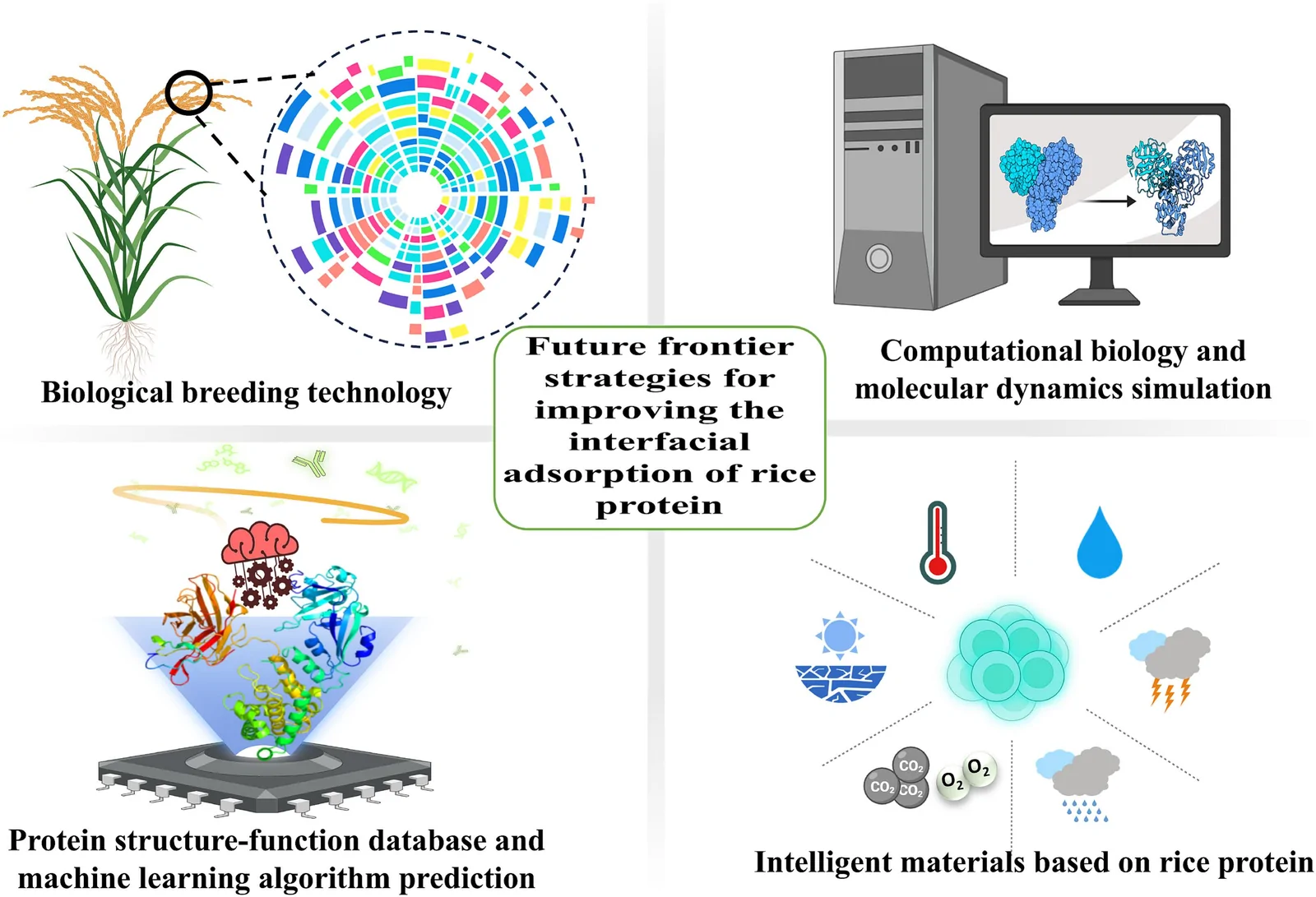
Future frontier strategies for improving the interfacial adsorption of rice protein (Macedo & Melo, 2024).

### 7.1 Biological breeding technology

Biological breeding shows great potential in improving the interfacial activity, emulsifying ability, and foaming properties of rice protein. By leveraging gene-editing technologies such as CRISPR-Cas9, we can precisely modify rice genes and directionally alter the amino acid sequence of rice protein. This is done to optimize its hydrophilicity and hydrophobicity, charge distribution, and conformational flexibility, thereby enhancing its interfacial activity at the source. This enables the design and expression of specific functional proteins.

### 7.2 Computational biology and molecular dynamics simulation

Computational biology combined with molecular dynamics simulations enables the prediction of self-assembly behaviors and interfacial adsorption conformations of rice protein across diverse amino acid sequences. This approach provides theoretical guidance for the rational design of protein nanostructures with tailored interfacial adsorption properties.

### 7.3 Construction of protein structure-function database and machine learning algorithm prediction

A systematic approach is employed to integrate high-throughput experimental data, construct a comprehensive protein structure–function database, and apply machine learning algorithms to predict the interfacial adsorption properties of modified rice proteins. This integrated strategy enables a high level of automation in the design and optimization of functional proteins.

### 7.4 Development of intelligent materials based on rice protein

Develop rice protein - based smart materials that can alter their interfacial adsorption behavior in response to external stimuli such as pH, temperature, ionic strength, and light. This can achieve intelligent control over the stability of emulsions or foams, meeting the requirements of specific food processing or storage.

## 8 Conclusion

Rice protein, a high-quality plant-based protein with low allergenicity, environmental sustainability, and balanced amino acid composition, holds significant promise as an emulsifier and foaming agent in the food industry. However, the inherent structural rigidity, low solubility, weak interfacial activity, and suboptimal charge properties of natural rice protein severely limit its widespread application in complex food systems. This review systematically summarizes effective approaches to enhance the interfacial adsorption behavior, emulsifying, and foaming properties of rice protein through physical, chemical, enzymatic, and composite modification strategies. By regulating the molecular structure, surface hydrophobicity, solubility, and charge distribution of rice protein, these modification methods significantly improve its ability to diffuse, spread, and form interfacial films at oil-water or air-water interfaces, thereby demonstrating broad application prospects in baked goods, dairy products, plant-based meat products, fortified and functional foods, and beverages.

Despite notable progress in improving the interfacial properties of rice protein, it is critical to fully account for the inherent differences in subunit composition and structure arising from variations in rice cultivars and raw material sources. This consideration is essential for precisely regulating interfacial adsorption behavior and optimizing applications in the food industry. Additionally, the translation from laboratory research to industrial application faces multiple challenges, including high equipment costs, technical limitations in large-scale production, potential impacts on product flavor and nutritional value, and uncertainty in performance within complex food matrices. Moreover, as a novel food ingredient, modified rice protein must meet stringent regulatory and safety assessment requirements.

Looking ahead, the optimization of rice protein's interfacial adsorption performance will trend toward more precise and intelligent regulation. This includes leveraging biobreeding technologies to optimize the amino acid sequence and structure of rice protein at the source, as well as using computational biology and molecular dynamics simulations to predict and design protein nanostructures with specific interfacial behaviors. Constructing protein structure-function databases integrated with machine learning algorithms will enable automated design and prediction of functional proteins, while developing rice protein-based smart materials that can respond to external stimuli to alter interfacial adsorption behavior. By integrating multidisciplinary approaches and deepening our understanding of the dynamic behavior of rice protein in complex food systems, we will overcome existing bottlenecks, fully unlock the enormous potential of rice protein as a sustainable, multifunctional food ingredient, and provide a solid theoretical foundation and technical support for the development of high-quality foods.

## CRediT authorship contribution statement

**Zhuangpeng Wang:** Writing – original draft, Resources, Formal analysis, Conceptualization. **Xinwen Huang:** Validation, Software, Resources. **Yu Zhao:** Validation, Software, Resources, Project administration. **Leiyan Wu:** Writing – review & editing, Funding acquisition.

## Declaration of competing interest

The authors declare that they have no known competing financial interests or personal relationships that could have appeared to influence the work reported in this paper.

## Data Availability

Data will be made available on request.
